# Supplementary material for: A brainstem to hypothalamic arcuate nucleus GABAergic circuit drives feeding
Source: Curr Biol. Author manuscript; Available in PMC 2025 Jan 23. (PMC7617324; doi:10.1016/j.cub.2024.02.074)
Supplement: Supplementary Material [file EMS202211-supplement-Supplementary_Material.zip › 1-s2.0-S0960982224002562-mmc1.pdf]

Current Biology, Volume *34*

Supplemental Information

A brainstem to hypothalamic arcuate nucleus GABAergic circuit drives feeding

Pablo B. Martinez de Morentin, J. Antonio Gonzalez, Georgina K.C. Dowsett, Yuliia Martynova, Giles S.H. Yeo, Sergiy Sylantyev, and Lora K. Heisler

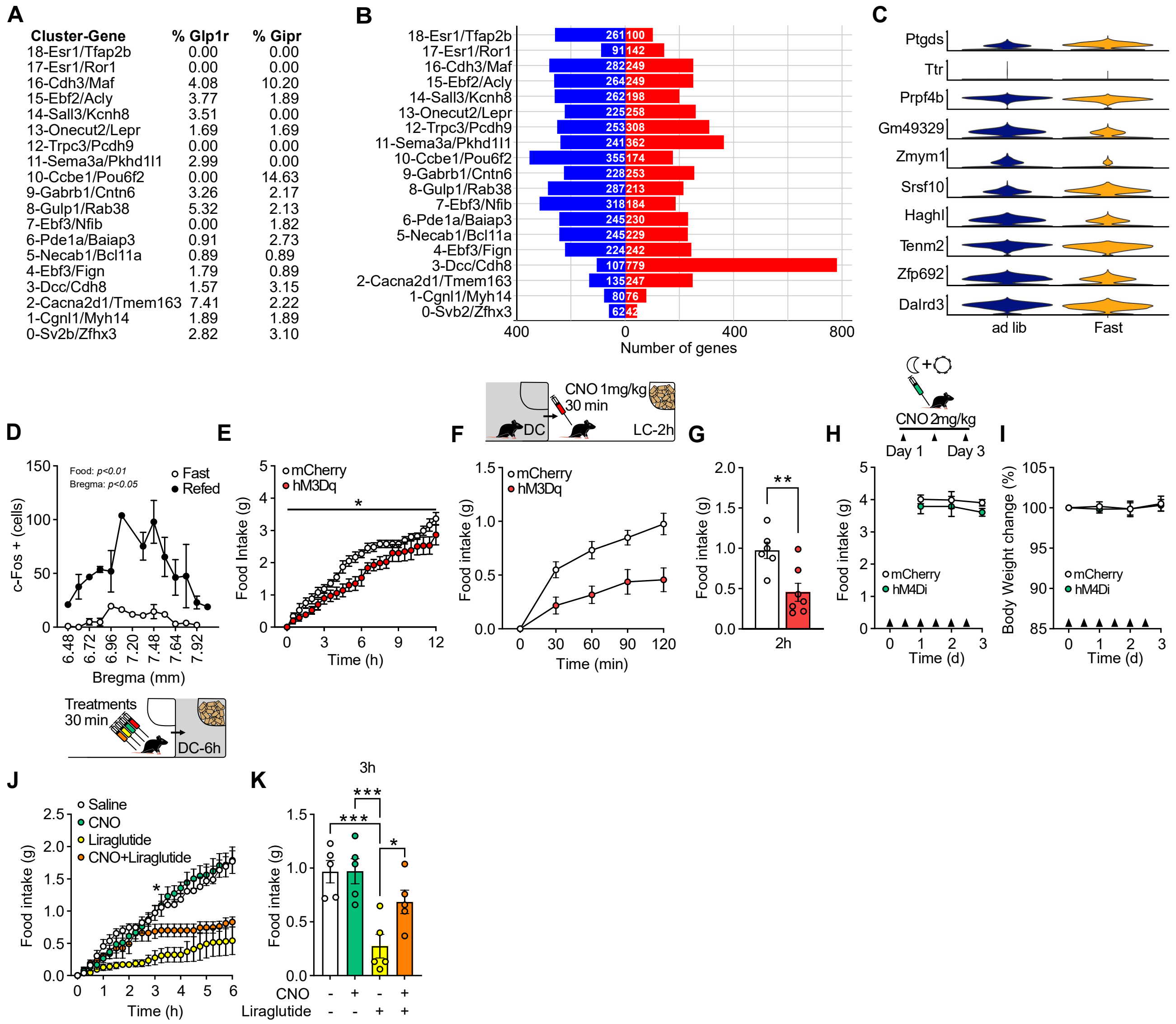

Figure S1. Characterization and function of GABA^{DVC} cells. Related to Figure 1.
(A) Proportion of GLP1-R and GIPR expression in each Slc32a1+ (GABA^{DVC}) cluster. (B) Number of genes upregulated (red) and downregulated (blue) in response to an overnight fast in each cluster. Genes included were significantly differentially regulated (p<0.05). (C) Top 10 genes differentially expressed in *ad libitum* fed vs fasted in Slc32a1+ neurons. (D) Quantification of total NTS c-Fos-expressing cells following 16h fasting and fasting+2h refeeding (n=3, two-way ANOVA Bregma level: $F_{(13,34)}$=3.16, p=0.004; Nutritional state: $F_{(1,4)}$=18.61, p=0.012). (E) 12h dark cycle food intake in GABA^{DVC}:hM3Dq vs control GABA^{DVC}:mCherry mice injected with clozapine-n-oxide (CNO). (F) Food intake following an overnight fast in GABA^{DVC}:hM3Dq mice compared to control GABA^{DVC}:mCherry (n=6, RM two-way ANOVA $F_{(1,11)}$=10.97, p=0.0069) treated with CNO (1 mg/kg, i.p.) and represented as (G) 2h total food intake (n=6/7, Unpaired t test, t(11)=3.392, p=0.006). (H) Food intake and (I) body weight change in GABA^{DVC}:hM4Di vs control GABA^{DVC}:mCherry mice injected twice daily with CNO (2 mg/kg, i.p.). (J-K) Food intake in GABA^{DVC}:hM4Di mice co-administered with CNO and Liraglutide (RM: ANOVA $F_{(3,12)}$=15.64, p=0.002, Bonferroni adjusted p=0.0004 Saline vs Liraglutide, P=0.0004 CNO vs Liraglutide and P=0.0263 Liraglutide vs Liraglutide+CNO). *: p<0.05, **: p<0.01, ***: p<0.001.

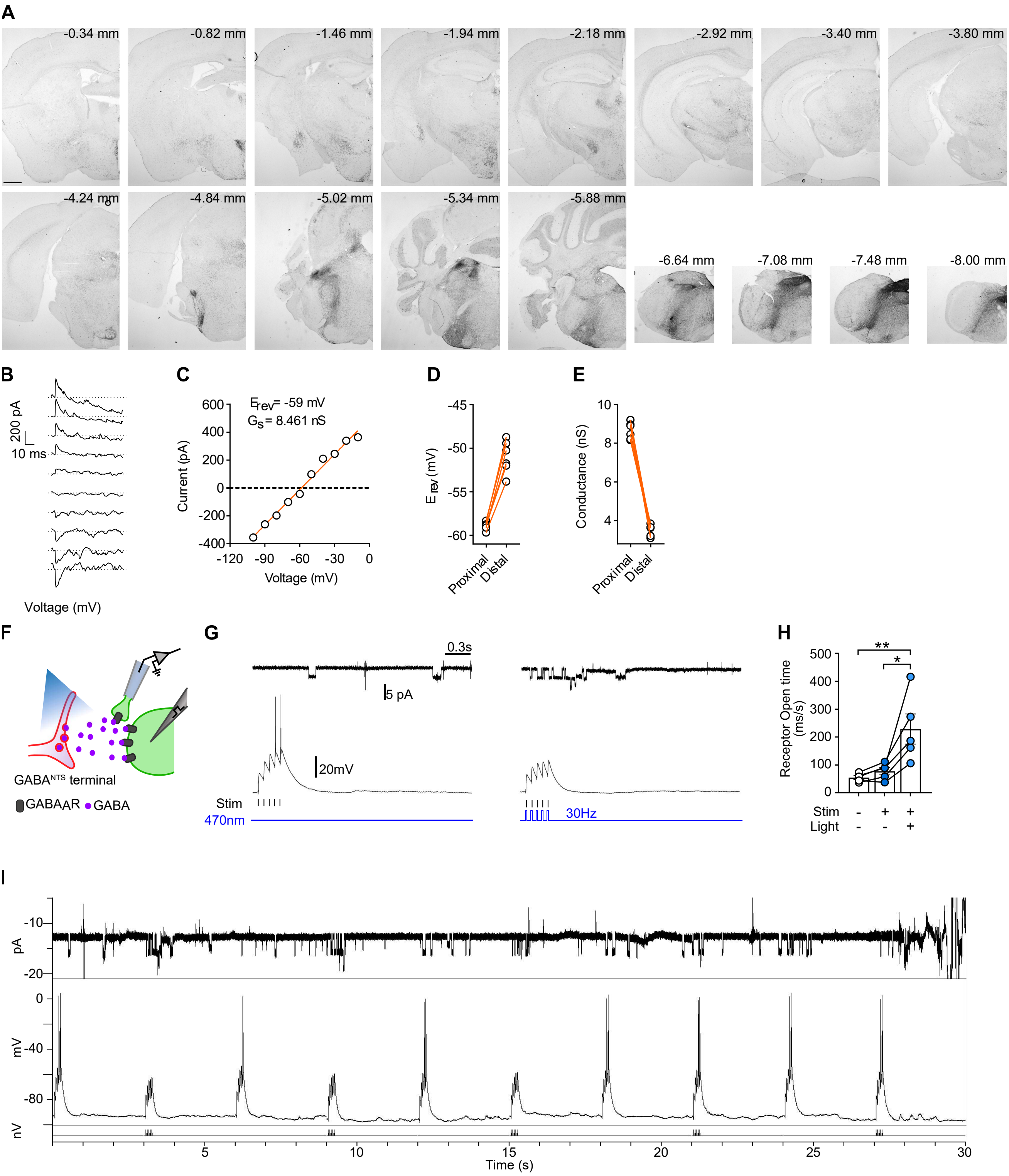

Figure S2. GABA^{DVC} projection pattern and CRACM additional analysis. Related to Figure 2.

(A) Representative photomicrographs (scalebar 100 µm) of ChR2-mCherry fibers in a serial rostro-caudal distribution of a *Vgat^{Cre}* mouse brain injected with AAV-DIO-ChR2-mCherry into the DVC. (B-E) A simulated voltage-clamp experiment in an anatomically realistic model of a medium spiny neuron, using the same voltage steps as those described for electrophysiology experiments (Figure 2E-G). The effect of optogenetic stimulation was modelled as a single GABAergic event taking place at each step. (C) Current amplitude was plotted against

voltage (as in Figure 2E-G), and a linear fit was used to estimate peak conductance G_s and reversal potential E_{rev}. (D-E) Simulated optogenetic activation of GABA inputs making synaptic contact only on dendrites close to the soma ("prox.", proximal dendrites) was compared to the same type of activation of GABA synapses connected only to dendrites far from the soma ("distal" dendrites) in six different medium spiny neuron models. The reversal potential for GABA in the model was set to -60 mV, and this is close to what was measured at the soma when GABA synaptic inputs were located on dendrites close to the soma. However, when GABA synapses were activated only on dendrites distant from the soma E_{rev}, was more positive than expected and conductance G_s was attenuated. (F) Diagram of outside-out patch technique coupled to a postsynaptic electrical stimulator. (G, left) Representative response of NPY^{hrGFP} cell from $Vgat^{Cre}::Npy^{hrGFP}$ mouse injected with AAV-DIO-ChR2-mCherry into the NTS subjected to series of five electrical stimuli, each evoking excitatory post-synaptic potential (EPSP) of sub-threshold amplitude. (G, right) The same cell with electrical stimuli coupled with 470 nm light burst. (H) Quantification of receptor opening time (n=5, two-way RM ANOVA $F_{(2,8)}=11.28$; p=0.0047, Bonferroni adjusted P=0.0072 NS vs SL and P=0.0157 S vs SL). (I) Representative response of a single NPY^{hrGFP} cell subjected to 10 series of five electrical stimuli, each evoking excitatory post-synaptic potential (EPSP) of sub-threshold amplitude coupled with 470nm light burst in $Vgat^{Cre}::Npy^{hrGFP}$ mouse injected with AAV-DIO-ChR2-mCherry into the NTS. NS: No stimulation; S: Stimulation; SL: Stimulation + Light. *: p<0.05, **: p<0.01.

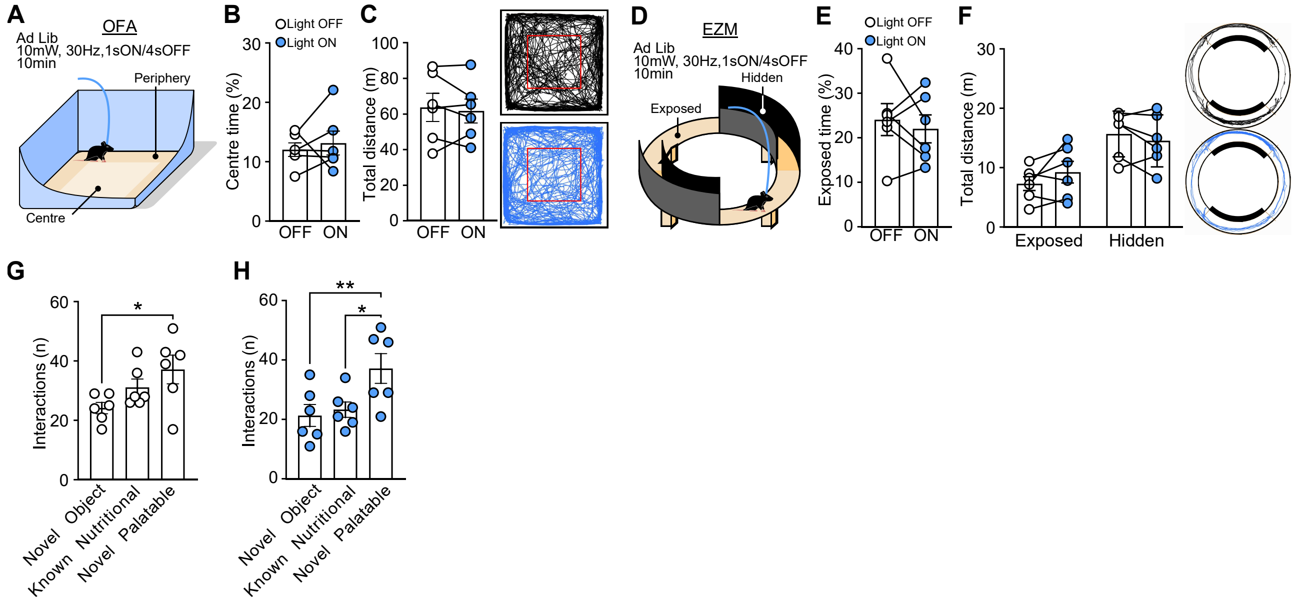

Figure S3. Optogenetic activation of GABA^{DVC}→ARC in VgatCre mice does not induce anxiety-like behavior. Related to Figure 3.

(A) Diagram illustrating open field arena (OFA) task. GABA^{DVC}→ARC optogenetic activation does not alter (B) time spent in the center or (C) distance travelled (representative trace of the movement) during the test. (D) Diagram illustrating elevated zero maze (EZM) task. GABA^{DVC}→ARC optogenetic activation does not alter (E) time spent in the exposed zone or (F) distance travelled in each zone (representative trace of the movement) during the test. (G-H) Quantification of number of interactions with a novel object, a known nutritional food item or a novel palatable food item in mice (G) without GABA^{DVC}→ARC stimulation (RM ANOVA ($F_{(2,10)}=4.993$; $p=0.0314$, Bonferroni adjusted $p=0.0306$ novel object vs novel palatable object) and (H) with GABA^{DVC}→ARC stimulation (RM ANOVA $F_{(2,10)}=10.71$; $p=0.0033$, Bonferroni adjusted $p=0.0051$ novel object vs novel palatable object; $p=0.0121$ known nutritional object vs novel palatable object). n=6 mice. *:$p<0.05$; **:$p<0.01$.
