# Supplementary material for: A brainstem to hypothalamic arcuate nucleus GABAergic circuit drives feeding
Source: Curr Biol. Author manuscript; Available in PMC 2025 Jan 23. (PMC7617324; doi:10.1016/j.cub.2024.02.074)
Supplement: Supplementary Material [file EMS202211-supplement-Supplementary_Material.zip › 1-s2.0-S0960982224002562-mmc4.pdf]

Article

Current Biology

A brainstem to hypothalamic arcuate nucleus GABAergic circuit drives feeding

Graphical abstract

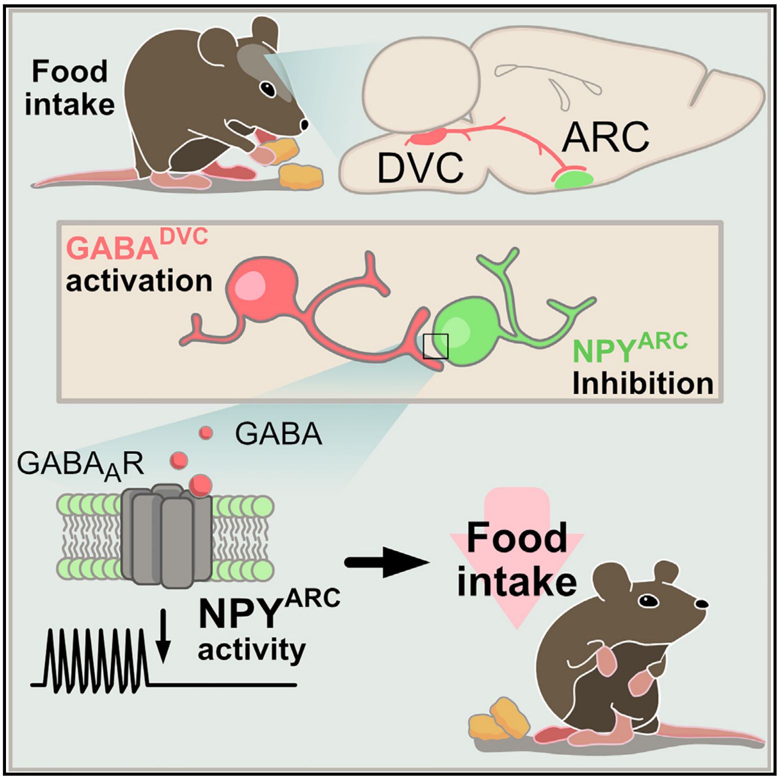

Highlights

- Dorsal vagal complex GABA (GABA^{DVC}) neurons are responsive to nutritional status
- Chemogenetic GABA^{DVC} neuron activation reduces food intake and body weight
- GABA^{DVC} projections to the hypothalamic arcuate nucleus (ARC) reduce feeding
- Optogenetic GABA^{DVC} → ARC stimulation inhibits orexigenic neuropeptide Y neurons

Authors

Pablo B. Martinez de Morentin, J. Antonio Gonzalez, Georgina K.C. Dowsett, Yuliia Martynova, Giles S.H. Yeo, Sergiy Sylantyev, Lora K. Heisler

Correspondence

p.demorentin@leeds.ac.uk (P.B.M.d.M.), s.sylantyev@abdn.ac.uk (S.S.)

In brief

Martinez de Morentin et al. identify GABAergic neurons in the dorsal vagal complex as a new player in the circuit governing feeding behavior and body weight.

Martinez de Morentin et al., 2024, Current Biology *34*, 1646–1656
April 22, 2024 © 2024 The Author(s). Published by Elsevier Inc.
https://doi.org/10.1016/j.cub.2024.02.074

CellPress

CellPress
OPEN ACCESS

Current Biology

Article

A brainstem to hypothalamic arcuate nucleus GABAergic circuit drives feeding

Pablo B. Martinez de Morentin,[1,2,5,7,*] J. Antonio Gonzalez,[1] Georgina K.C. Dowsett,[3] Yuliia Martynova,[1] Giles S.H. Yeo,[3] Sergiy Sylantyev,[1,4,*] and Lora K. Heisler[1,6]

[1]The Rowett Institute, University of Aberdeen, Ashgrove Road W, Aberdeen AB25 2ZD, UK
[2]School of Biomedical Sciences, Faculty of Biological Sciences, University of Leeds, Woodhouse LS2 9JT, UK
[3]MRC Metabolic Diseases Unit, Institute of Metabolic Science, University of Cambridge, Addenbrooke's Hospital, Cambridge CB2 0QQ, UK
[4]Odesa National Mechnikov University, Biological Department, 2 Shampansky Ln., Odesa 65015, Ukraine
[5]X (formerly Twitter): @pablomorentin
[6]X (formerly Twitter): @LoraHeisler
[7]Lead contact
*Correspondence: p.demorentin@leeds.ac.uk (P.B.M.d.M.), s.sylantyev@abdn.ac.uk (S.S.)
https://doi.org/10.1016/j.cub.2024.02.074

SUMMARY

The obesity epidemic is principally driven by the consumption of more calories than the body requires. It is therefore essential that the mechanisms underpinning feeding behavior are defined. Neurons within the brainstem dorsal vagal complex (DVC) receive direct information from the digestive system and project to second-order regions in the brain to regulate food intake. Although γ-aminobutyric acid is expressed in the DVC ($GABA^{DVC}$), its function in this region has not been defined. In order to discover the unique gene expression signature of $GABA^{DVC}$ cells, we used single-nucleus RNA sequencing (Nuc-seq), and this revealed 19 separate clusters. We next probed the function of $GABA^{DVC}$ cells and discovered that the selective activation of $GABA^{DVC}$ neurons significantly controls food intake and body weight. Optogenetic interrogation of $GABA^{DVC}$ circuitry identified $GABA^{DVC}$ → hypothalamic arcuate nucleus (ARC) projections as appetite suppressive without creating aversion. Electrophysiological analysis revealed that $GABA^{DVC}$ → ARC stimulation inhibits hunger-promoting neuropeptide Y (NPY) neurons via GABA release. Adopting an intersectional genetics strategy, we clarify that the $GABA^{DVC}$ → ARC circuit curbs food intake. These data identify $GABA^{DVC}$ as a new modulator of feeding behavior and body weight and a controller of orexigenic NPY neuron activity, thereby providing insight into the neural underpinnings of obesity.

INTRODUCTION

Obesity represents a key challenge to human health and is primarily due to the consumption of calories in excess of the body's energy requirements. Eating is a complex behavior that not only depends on the basic energy demands at a cellular and organismal level but also the integration of internal and environmental cues, the reward value of food, motivation, and conditioning behavior.[1,2] The aim of the present study was to probe neurocircuitry regulating feeding and body weight with the objective of uncovering critical energy homeostasis circuitry.

One of the primary nodes for the integration of energy-related information from the periphery to the brain is the dorsal vagal complex (DVC).[3] This region includes the area postrema, the nucleus of the solitary tract (NTS), and the dorsal motor nucleus of the vagus. The DVC contains a heterogeneous population of neurons responsive to energy status.[4–6] Subpopulations influencing energy balance include leptin receptor (LEPR),[7,8] calcitonin receptor (CALCR),[9] glucagon-like peptide-1 receptor (GLP-1R),[10–12] preproglucagon (PPG),[13] tyrosine hydroxylase (TH),[14–16] cholecystokinin (CCK),[17,18] prolactin-related peptide,[19–21] and proopiomelanocortin (POMC).[22,23] Recent studies using DVC single-nucleus RNA sequencing (Nuc-seq) provide an expression map of these cellular populations, and most were revealed to be glutamatergic.[4,24] However, very little is known about the role of DVC inhibitory GABA-releasing clusters ($GABA^{DVC}$) in energy homeostasis. A report in rats indicated that a subpopulation of DVC GLP-1R-expressing neurons releasing GABA are necessary mediators for the anorectic effects of the obesity medication liraglutide.[11] Therefore, DVC GABAergic cells represent an intriguing and understudied population of hindbrain cells and are the focus of the present study.

In the regulation of feeding behavior, one of the most widely studied projections from the NTS subregion of the DVC is to the hypothalamus.[14,17,25–28] However, whether the hypothalamic arcuate nucleus (ARC) receives direct GABAergic inhibitory control from the NTS is not known. Within the ARC, there is a subpopulation of potent orexigenic neurons expressing both agouti-related peptide and neuropeptide Y (AgRP/NPY).[29–32] We hypothesized that $GABA^{DVC}$ neurons significantly regulate feeding and body weight and project to and inhibit key food-intake-stimulating AgRP/NPY cells.

 © 2024 The Author(s). Published by Elsevier Inc.
This is an open access article under the CC BY license (http://creativecommons.org/licenses/by/4.0/).

CellPress
OPEN ACCESS

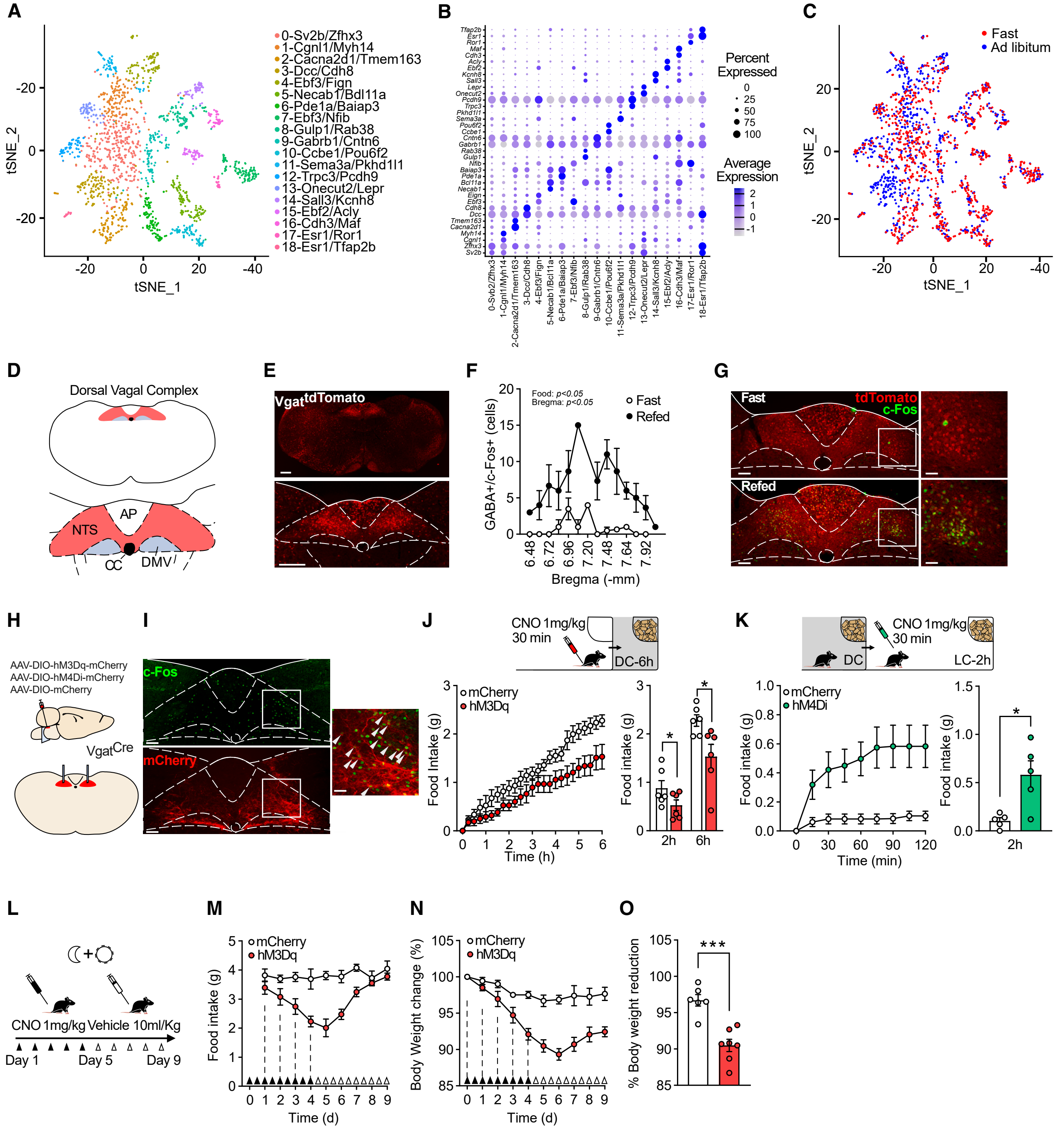

Figure 1. GABA^{DVC} activation reduces food intake and body weight

(A) tSNE plot of *Slc32a1*$^{+}$ neurons in the DVC colored by cluster.

(B) Dot plot showing scaled expression of marker genes for each of the 19 *Slc32a1*$^{+}$ clusters.

(C) tSNE plot of the *Slc32a1*$^{+}$ nuclei colored by nutritional status.

(D) Schematic of the DVC with diagram showing AP, NTS, and DMV subregions.

(E) Representative photomicrograph (scale bars, 500 μm) of GABAergic cell distribution in the medial brainstem (top) and in the DVC (bottom) using *Vgat*$^{Cre:tdTom}$ mice.

(F) Quantification of GABAergic cells expressing c-Fos following 16 h fasting and fasting + 2 h refeeding (n = 3, two-way ANOVA, bregma level: $F_{(13,34)} = 2.63$; p = 0.012; nutritional state: $F_{(1,4)} = 8.69$; p = 0.042).

(G) Representative micrograph (scale bars, 200 μm) of *Vgat*^{tdTom} cells (red) expressing c-Fos (green) in mice fasted 16 h (top) and re-fed for 2 h (bottom) and magnifications (scale bars, 100 μm).

(legend continued on next page)

RESULTS

Profile of GABA^{DVC} cells via Nuc-seq

To provide the first detailed characterization of GABA^{DVC} neurons, we used a Nuc-seq dataset of the mouse DVC.[4] Neuronal cells expressing transcripts for *Slc32a1* (solute carrier family 32 member 1 or vesicular GABA transporter [*Vgat*]) were extracted and re-clustered, consisting of 1,847 neurons that formed 19 clusters (Figure 1A; see Data S1 for more information). Of these clusters, 2-*Cacna2d1/Tmem163* expressed Vglut2 transcripts and lower levels of *Gad1* and *Gad2*. Low expression of classical neurotransmitter-related genes was found in these GABAergic neuronal clusters (Figure 1B). Adipocyte hormone leptin receptor (*Lepr*) expression was identified in clusters 13-*Onecut2/Lepr* and 15-*Ebf2/Acly* (Figure 1B). Low-level expression of incretin receptors GLP-1R and gastric inhibitory polypeptide receptor (GIPR) was evident in several clusters (Figure S1A). Differential gene expression analysis was performed on each cluster to identify the effects of an overnight fast on transcript expression in DVC GABAergic (*Slc32a1*+) neurons (Figures 1C and S1B; see Data S2 for more information). Cluster 3-*Dcc/Cdh8* displayed significant upregulation in transcript expression in response to the fast, however, 93.7% of this cluster originates from *ad libitum*-fed animals. Significantly differentially regulated genes in *Slc32a1*$^+$ neurons can be found in Figure S1C. These data indicate minimal overlap with specific populations of DVC neurons that have been previously described with regard to energy balance.

GABA^{DVC} cells are sensitive to energy status and modulate food intake

We next examined the function of GABA^{DVC} neurons. Visualization of GABA^{DVC} neurons was facilitated by crossing *Vgat-ires-Cre* mice with *Rosa26-tdTomato*$^{fl/fl}$ reporter mice (*Vgat*^{tdTom}, Figures 1D and 1E). To establish whether these neurons are sensitive to energy status, expression levels of the *in vivo* neuronal activity marker c-Fos[33] were quantified in mice that were overnight fasted and in mice that were refed for 2 h after overnight fasting. Refed mice showed significantly more c-Fos in the NTS portion of the DVC (Figure S1D), and specifically within NTS GABAergic cells, compared with fasted mice (Figures 1F and 1G). This suggests that GABA^{DVC} cells are responsive to refeeding.

To determine whether GABA^{DVC} neurons have a role in the control of food intake, we used chemogenetic designer receptors exclusively activated by designer drugs (DREADD) to manipulate GABA^{DVC} cellular activity.[34] Specifically, transduction with AAVs delivering Cre-dependent expression of stimulatory receptors using AAV8-hSyn-DIO-hM3DGq (GABA^{DVC}:hM3Dq mice), inhibitory receptors using AAV8-hSyn-DIO-hM4DGi-mCherry (GABA^{DVC}:hM4Di mice), or control using AAV8-hSyn-DIO-mCherry (GABA^{DVC}:mCherry) were bilaterally injected into the DVC of *Vgat*^{Cre} mice (Figure 1H). This allowed modulation of GABA^{DVC} neuron activity through administration of the designer drug clozapine-N-oxide (CNO). Treatment with CNO in GABA^{DVC}:hM3Dq mice induced strong c-Fos expression in the NTS (Figure 1I). We next assessed the effect of the modulation of GABA^{DVC} on food intake in mice under different scenarios. In *ad libitum* fed mice, GABA^{DVC}:hM3Dq chemogenetic activation of GABA^{DVC} cells significantly reduced overall food intake during the dark cycle compared with control GABA^{DVC}:mCherry siblings (Figures 1J and S1E). Similarly, CNO cells' activation reduced food intake in hunger-induced fasted GABA^{DVC}:hM3Dq mice compared with control GABA^{DVC}:mCherry mice (Figures S1F and S1G). Conversely, chemogenetic inhibition of GABA^{DVC} cell in satiated GABA^{DVC}:hM4Di mice during the light cycle significantly increased food intake when compared with satiated littermate GABA^{DVC}:mCherry controls (Figure 1K).

Given the potent reduction of food intake produced by the activation of GABA^{DVC} neurons, we examined whether prolonged activation impacted body weight. Twice-daily administration of CNO (Figure 1L) in GABA^{DVC}:hM3Dq mice induced a significant reduction in daily food intake (Figure 1M) and progressive weight loss (Figures 1N and 1O). Although feeding returned to baseline levels 72 h after the final CNO administration, body weight remained significantly lower in hM3Dq-expressing mice compared with GABA^{DVC}:mCherry controls (Figure 1N). In contrast, prolonged GABA^{DVC} neuronal inhibition did not induce greater food intake (Figure S1H) nor changes in body weight (Figure S1I) in GABA^{DVC}:hM4Di mice compared with littermate GABA^{DVC}:mCherry controls. These results indicate that selective activation of GABA^{DVC} neurons is sufficient to promote sustained reductions in energy intake and body weight.

A recent report in rats indicated that inhibition of neurons expressing the GABA-producing enzyme glutamate decarboxylase (GAD) in the NTS partially blunted the anorectic effect of the GLP1-R agonist liraglutide.[11] These data suggest that GABA^{NTS} neurons contribute to the therapeutic effects of GLP1-R agonists in rats. However, GLP-1R expression in rats and mice differs, especially with regard to receptor density.[35–37]

(H) Schematic of AAV-DIO-hM3Dq/hM4Di/mCherry (see STAR Methods viral vectors) infused into NTS of *Vgat*^{Cre} mice.

(I) Representative photomicrograph, right, (scale bars, 200 μm) of c-Fos (green) expression in GABA^{DVC}:hM3Dq-mCherry (red)-expressing cells in the NTS of *Vgat*^{Cre} mice treated with clozapine-n-oxide (CNO, 1 mg/kg intraperitoneally [i.p.]) and magnification, left (scale bar, 100 μm). White arrows indicate double labelled cells.

(J) 6 h cumulative (left) and 2 h and 6 h total (right) food intake ($n = 6$, $t(11) = 2.663$, and $p = 0.0238$) in GABA^{DVC}:hM3Dq CNO-injected mice compared with GABA^{DVC}:mCherry.

(K) 2 h cumulative and (J) 2 h total food intake ($n = 5$, $t(8) = 3.205$, and $p = 0.0125$) in GABA^{DVC}:hM4Di CNO-injected mice compared with GABA^{DVC}:mCherry mice.

(L–O) (L) 10-day treatment protocol schematic. Twice-daily CNO treatment significantly reduced (M) food intake (RM two-way ANOVA $F_{(1,11)} = 20.57$; $p = 0.0008$), (N) body weight (RM two-way ANOVA ($F_{(1,11)} = 21.96$; $p = 0.0007$), and (O) body weight change in GABA^{DVC}:hM3Dq mice compared with control GABA^{DVC}:mCherry mice ($t(11) = 5.252$, $p = 0.0003$).

(C and D) n = 4/group, (H–K) n = 6, (M–O) n = 6 GABA^{DVC}:mCherry and n = 7 GABA^{DVC}:hM3Dq. Data are represented as mean ± SEM. *$p < 0.05$, **$p < 0.01$, and ***$p < 0.001$. AP, area postrema; CC, center canal; DMV, dorsal motor nucleus of the vagus; NTS, nucleus of the solitary tract; tSNE, t-distributed stochastic neighbor embedding.

See also Figure S1 and Data S1 and S2 for more information.

We therefore assessed the feeding response of liraglutide in GABA^{DVC}:hM4Di-expressing mice. Chemogenetic inhibition of GABA^{DVC} neurons partially blunted the acute anorectic effect of liraglutide (Figures S1J and S1K). This provides support for the notion that GABA^{DVC} neurons contribute to the anorectic effects of liraglutide in mice.

Optogenetic stimulation of GABA^{DVC} terminals inhibits NPY^{ARC} cells

To clarify the circuitry through which GABA^{DVC} neurons influence feeding and body weight, we used Channelrhodopsin 2 (ChR2)-assisted circuit mapping (CRACM).[38,39] Specifically, transduction with AAVs delivering Cre-dependent expression of AAV2-EF1a-DIO-ChR2(E123T/T159C)-mCherry were bilaterally injected into the DVC of *Vgat*^{Cre} mice (Figure 2A) and projection patterns were analyzed. GABA^{DVC} cells project to hypothalamic subregions including the ARC, paraventricular nucleus (PVH), dorsomedial nucleus (DMH), and other extra-hypothalamic regions (Figure S2A). Within the ARC, we observed a dense array of projections (Figure 2B). This identified the ARC as a candidate second-order region involved in GABA^{DVC} neuronal control of food intake and body weight.

Fasting increases the activity of AgRP/NPY neurons[32] and direct AgRP/NPY neuron activation induces robust feeding.[40,41] Because GABA-releasing neurons are the main inhibitory network in the brain,[42] we hypothesized that GABA^{DVC} cells projecting to the ARC would target AgRP/NPY neurons to decrease food intake. To interrogate this potential GABA^{NTS} → NPY^{ARC} circuit, *Vgat*^{Cre} mice were crossed with *Npy*^{hrGFP} mice (*Vgat*^{Cre}*::Npy*^{hrGFP}) and bilaterally injected with AAV2-EF1a-DIO-ChR2 (E123T/T159C)-mCherry into the DVC. We observed that a subset of NPY^{hrGFP} cell bodies were surrounded by mCherry-containing fibers (Figure 2C). In contrast, terminals were not found surrounding neurons expressing another neuropeptide involved in the regulation of food intake, POMC neurons[43] (Figure 2D).

We next investigated whether this anatomical connectivity produced functional interactions between GABA^{DVC} terminals and NPY and POMC neurons in the ARC. To do this, we used *Vgat*^{Cre}*::Npy*^{hrGFP} mice bilaterally infused into the DVC with AAV2-EF1a-DIO-ChR2(E123T/T159C)-mCherry. We also crossed *Vgat*^{Cre} mice the *Pomc*^{DsRed} reporter line (*Vgat*^{Cre}*::Pomc*^{dsRED}) and injected bilaterally into the DVC with AAV2-EF1a-DIO-ChR2(E123T/T159C)-YFP. Photo-stimulation of ChR2-containing axon terminals from GABA^{DVC} neurons produced robust synaptic responses in 14% of NPY cells in the ARC of *Vgat*^{Cre}*::Npy*^{hrGFP} mice but not in POMC cells using *Vgat*^{Cre}*::Pomc*^{DsRed} mice (Figure 2E). The rapid synaptic currents triggered in NPY^{ARC} cells by the optical stimulation changed polarity near the equilibrium potential for chloride (Figures 2F and 2G), as expected from ionotropic GABA receptors. Light-induced currents were unexpectedly small, and their reversal potential was more positive than that expected for GABA-activated currents (about −60 mV) (Figures 2H–2J). These effects could be explained by voltage- and space-clamp errors[44] and in turn suggest that perhaps GABA^{DVC} terminals reach NPY^{ARC} cells at dendrites distant from the soma. Although it is not possible to directly demonstrate that this is the case, we tested whether this reasoning was justified by simulating the optogenetic activation of GABA synaptic events at distal vs. proximal dendrites in a model neuron. Using a predictive neuronal model,[45] we found that GABAergic postsynaptic currents become progressively smaller, with their reversal potential progressively more positive the further the GABA inputs are from the soma (Figures S2B–S2E).

Next, we tested whether activation of GABA receptors at NPY^{ARC} cells is accompanied by release of GABA from GABA^{DVC} ChR2-containing terminals. We first stimulated ChR2-mCherry-expressing GABA^{DVC} fibers in the ARC and performed a "sniffer patch" experiment registering GABA$_A$R single-channel openings above the ARC NPY^{hrGFP} cells contacted by GABA^{DVC} fibers (Figure 2K). In this experiment, the GABA$_A$R response was isolated with a specific cocktail of antagonists (see STAR Methods). This provides semi-quantitative monitoring of extracellular levels of GABA.[46] A burst of light directed to GABA^{DVC} ChR2-containing terminals in the ARC induced single-channel openings in the membrane patch in control conditions, and the currents had amplitudes comparable with GABAergic inhibitory currents[46] (Figure 2L, left). Application of GABA$_A$R competitive antagonist gabazine reversibly blocked the single-channel openings (Figure 2L, middle and right). The same pattern of receptor opening time was observed in all assessed patches (Figure 2M). This suggested that postsynaptic NPY^{ARC} cells are sensitive to GABA^{DVC} presynaptic release of GABA.

To test whether NPY^{ARC} cells were inhibited by the release of GABA from GABA^{DVC} terminals, we designed a protocol of additive subthreshold electrical stimuli. This method is designed to evoke an action potential after 5 stimuli. In addition, we coupled a 470-nm light burst to the same trigger (Figure S2F). We then alternated a sequence of electrical stimuli and electrical stimuli with light burst. The electrical stimulation evoked an action potential(s) (Figure S2G, left), followed by low-frequency or no single-channel openings in the sniffer patch. When we coupled the electrical stimulation with the light burst, the occurrence of actions potentials was blocked and accompanied by single-channel openings in a sniffer patch (Figure S2G, right). This happened in all cells patched (Figure S2H). These openings resembled GABA$_A$R openings illustrated in Figure 2L. Subsequent stimulations showed a decrease in channel opening intensity, suggesting a depletion of GABA stores from the presynaptic terminal, which was eventually insufficient to prevent the action potential (Figure S2I). These results provide strong evidence that GABA^{DVC} fibers inhibit NPY^{ARC} cells due to the release of the fast neurotransmitter GABA.

GABA^{DVC} → ARC optogenetic stimulation reduces feeding and is not aversive

Given the dense fiber projection pattern of DVC GABAergic cells to the ARC, and that its activation inhibited NPY^{ARC} neurons, we interrogated whether this circuit is sufficient to influence food intake. To investigate this, AAV2-EF1a-DIO-ChR2(E123T/T159C)-mCherry was bilaterally infused into the DVC of *Vgat*^{Cre} mice and an optic fiber was placed above the ARC (Figure 3A). Food intake was measured both without and with ARC photostimulation prior to the onset of the dark cycle (Figures 3B and 3C). Light stimulation of GABA^{DVC} → ARC terminals induced

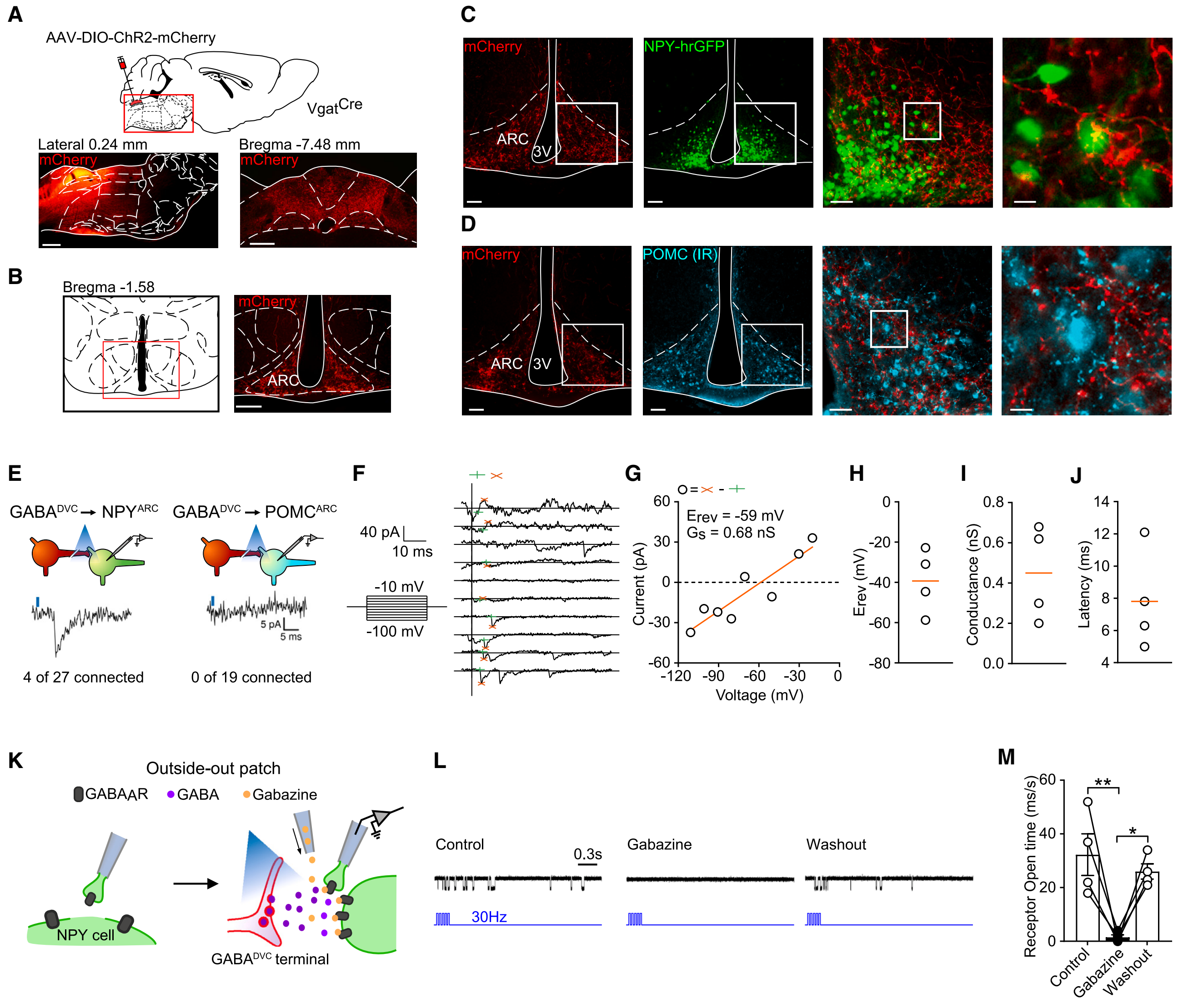

Figure 2. GABA^{DVC} projections to the ARC release GABA at NPY^{ARC} neurons

(A and B) (A) Illustration depicting DVC injection of AAV2-EF1a-DIO-ChR2(E123T/T159C)-mCherry (top) and representative photomicrograph (scale bars, 1 mm) of injection (bottom) in *Vgat^{Cre}* mice and (B) ChR2-mCherry-containing fibers in the ARC (scale bars, 200 μm).

(C and D) (C) Representative photomicrograph (scale bars, 100 μm) and magnifications (scale bars, 50 and 10 μm) of ChR2-mCherry fibers in the ARC close to NPY^{hrGFP}-expressing cells but not (D) POMC-expressing cells in *Vgat^{Cre}* mice bilaterally injected with AAV-DIO-ChR2-mCherry.

(E–J) CRACM study in *Vgat^{Cre}*::*Npy^{hrGFP}* and *Vgat^{Cre}*::*Pomc^{DsRed}* mice injected with AAV-DIO-ChR2-mCherry into the DVC. (E) Representative membrane potential response of NPY^{hrGFP} and POMC^{DsRed} cells after light stimulation (blue shading) of GABA^{DVC} → ARC mCherry-containing terminals. 4/27 NPY^{hrGFP} cells and 0/19 cells POMC^{DsRed} cells responded. (F) Representative membrane current in NPY^{hrGFP} cell recorded during a voltage clamp experiment from −100 mV to −10 mV in 10-mV steps. (vertical line, light pulse; green cross, base line; red cross, peak value.) (G) Representative light-induced postsynaptic current during voltage clamp experiment showing peak conductance, G_s, and reversal potential, E_{rev}. (H) Individual values and median of (H) E_{rev}, (I) conductance, G_s and (J) latency of all responsive cells.

(K) Diagram of outside-out patch technique in *Vgat^{Cre}*:*Npy^{hrGFP}* mice bilaterally injected with AAV-DIO-ChR2-mCherry into the DVC.

(L) Representative channel opening recordings of control (left), gabazine (middle), and washout (right) recordings.

(M) Quantification of receptor opening time (n = 4, RM two-way ANOVA $F_{(2,6)} = 16.14$; $p = 0.0039$, Bonferroni adjusted $p = 0.0051$ control vs. gabazine and $p = 0.0156$ gabazine vs. washout. Data in (M) are expressed as mean ± SEM. *$p < 0.05$; **$p < 0.01$.

See also Figure S2.

an acute inhibition of food intake, which lasted 60 min from food presentation, as compared with the same mice without light stimulation (Figures 3D and 3E).

The DVC has been proposed to be a key region modulating food intake reduction associated with aversive states.[47] To assess whether GABA^{DVC} → ARC activation produces aversion or negative valence,[48,49] we evaluated the existence of passive avoidance behavior using an adapted real-time place preference (RTPP) task.[17,50,51] Specifically, in a two-sided open arena, light stimulus was coupled to one of the sides (Figure 3F).

CellPress
OPEN ACCESS

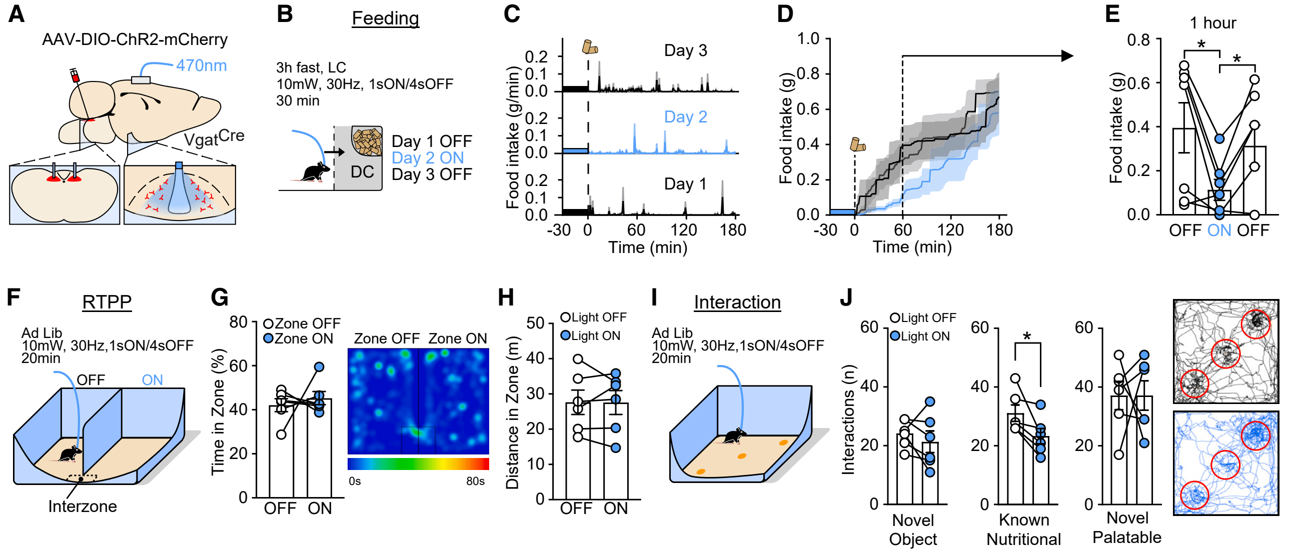

Figure 3. Activation of GABA^{DVC} → ARC projections reduce food intake without inducing aversion

(A) Schematic of DVC injection of AAV2-EF1a-DIO-ChR2(E123T/T159C)-mCherry in *Vgat^{Cre}* mice and optic fiber placement for *in vivo* stimulation of GABA^{NTS} → ARC terminals.

(B) Protocol of stimulation.

(C–E) (C) Representative 1 min resolution time plot of 3 h food intake measurement. GABA^{DVC} → ARC stimulation significantly reduces (D) cumulative 3 h food intake and (E) 60 min food intake (n = 7, day 1 [OFF] vs. day 2 [ON] t test $t(6) = 3.035$, $p = 0.0229$ and day 2 [ON] vs. day 3 [OFF] $t(6) = 3.283$, $p = 0.0168$).

(F–H) (F) Diagram illustrating real-time place preference (RTPP) task. GABA^{DVC} → ARC does not alter (G) time spent (representative heat map of time) or (H) distance traveled in stimulated and non-stimulated zones.

(I) Diagram illustrating an object interaction task.

(J) Interactions with novel, known nutritional, or novel palatable objects and a representative path track around each object (red circle). GABA^{DVC} → ARC stimulation reduced interaction with known nutritional item (n = 6, $t(5) = 3.043$, and $p = 0.0287$). Data are expressed as individual values and as mean ± SEM, n = 6 mice. *$p < 0.05$.

See also Figure S3.

GABA^{DVC} → ARC stimulation or lack of stimulation did not produce a place preference (Figure 3G) and mice traveled a similar distance in both sides of the arena (Figure 3H). These findings indicate that GABA^{DVC} → ARC stimulation does not produce negative valence or aversion.

In addition to influencing homeostatic feeding, modulation of AgRP/NPY cells has also been reported to elicit anxiety-like behaviors influencing exploration and foraging, which impacts food consumption.[52–54] To test whether the activation of the GABA^{DVC} → ARC circuit produced anxiety-related behavior, *Vgat^{Cre}* mice injected into the DVC with AAV2-EF1a-DIO-ChR2(E123T/T159C)-mCherry, with an optic fiber placed above the ARC, were assessed in an open-field arena (OFA) and an elevated zero maze (EZM) task (Figures S3A and S3D). GABA^{DVC} → ARC stimulation did not alter the time mice spent in the center of the OFA (Figure S3B). Likewise, mice displayed similar ambulatory patterns and traveled comparable distance during the OFA test with and without GABA^{DVC} → ARC stimulation (Figure S3C). Consistent with the OFA data, GABA^{DVC} → ARC stimulation did not alter either the time (Figure S3E) or distance traveled in the exposed zones of the EZM (Figure S3F). These results provide evidence that stimulation of the GABA^{DVC} → ARC circuit impacts feeding without altering other behavioral states such as anxiety-like behavior.

We postulated that because our model involved fast neurotransmission rather than long-term release of neuropeptides, the reduction in food intake that we observed could begin by reducing the interaction with the nutritional cue at first instance. To investigate this, we designed a task where mice tested in the tasks above would first be allowed to explore an empty arena and then three items would be presented at the same time: an inedible object, a novel palatable food item, and a known nutritional food item (Figure 3I). The number of interactions with each item was measured with and without GABA^{DVC} → ARC stimulation. GABA^{DVC} → ARC stimulation did not induce changes in the interaction with the novel object (Figure 3J), further suggesting that GABA^{DVC} → ARC does not induce anxiety-like behavior. The number of interactions with the novel palatable food item was increased under both non-stimulation and stimulation trials (Figures S3G and S3H). However, we found that under optical stimulation, mice had less interactions with the known nutritional item (Figures S3G and S3H), suggesting that GABA^{DVC} → ARC activation reduces hunger. Taken together, these findings indicate that GABA^{DVC} → ARC stimulation decreases feeding without inducing aversion or anxiety.

GABA^{DVC} → ARC neuron activation reduces food intake

We next used a two-virus intersectional approach[55,56] to provide a more detailed characterization of GABA^{DVC} → ARC activation in energy homeostasis. *Vgat^{Cre}* mice were bilaterally injected with AAV-expressing flippase recombinase (FlpO)

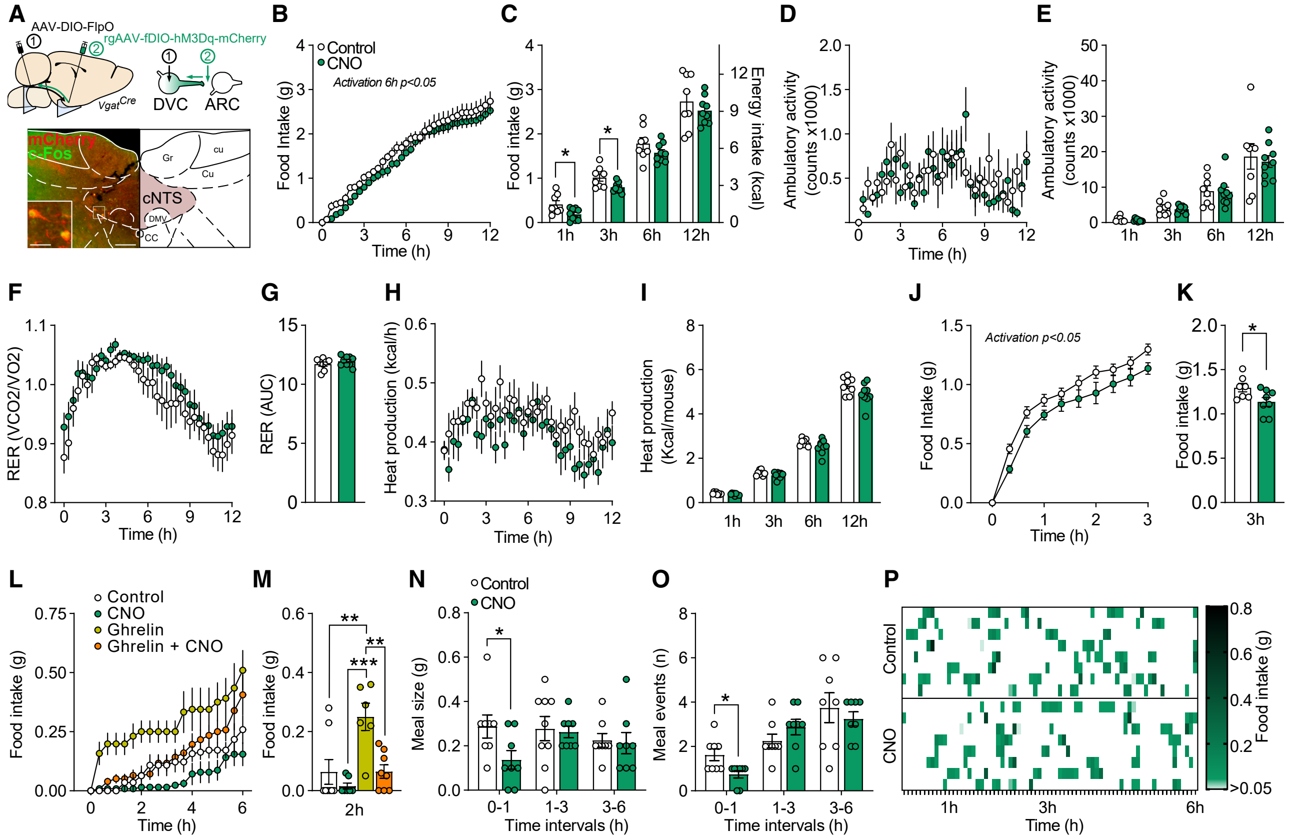

Figure 4. Selective chemogenetic activation of GABA^{DVC} → ARC neurons supresses feeding

(A) Diagram illustrating the two-virus intersectional strategy to express hM3Dq only in GABA^{DVC} neurons projecting to the ARC (GABA^{DVC} → ARC:hM3Dq mice). Representative photomicrograph (scale bars, 500 and 50 μm) depicting mCherry-expressing cells in the caudal NTS (red), c-Fos (green), and co-labeled (yellow) following treatment with clozapine-n-oxide (CNO, 1 mg/kg i.p.).

(B and C) (B) CNO significantly reduced cumulative food intake over 6 h (0–6 h $F_{(1,15)} = 7.418$, $p = 0.0157$), (C) 1 h ($t(15) = 2.648$, $p = 0.0183$), and 3 h ($t_{(15)} = 2.568$, $p = 0.0214$) compared with saline in GABA^{DVC} → ARC:hM3Dq mice.

(D–K) (D) CNO did not alter 12 h or (E) 1, 3, 6, or 12 h quantification of ambulation; (F) 12 h RER or (G) AUC quantification; (H) 12 h or (I) 1, 3, 6, and 12 h heat production per mouse compared with saline in GABA^{DVC} → ARC:hM3Dq mice. (J and K) CNO significantly reduced 3 h food intake following overnight fasting compared with control saline treatment (J, two-way ANOVA $F_{(1,13)} = 6.139$, $p = 0.0277$; and K, $t(13) = 2.320$, $p = 0.0372$) in GABA^{DVC} → ARC:hM3Dq mice.

(L and M) CNO attenuated ghrelin hyperphagia over 2 h (RM ANOVA $F_{(3,16)} = 11.12$, $p = 0.0003$, Bonferroni adjusted $p = 0.0042$ control vs. ghrelin, $p = 0.0003$ CNO vs. ghrelin, and $p = 0.002$ ghrelin vs. ghrelin+CNO) in GABA^{DVC} → ARC:hM3Dq mice.

(N–P) (N) CNO reduced meal size ($t(14) = 2.256$, $p = 0.0406$) and (O–P) decreased meal number ($t(14) = 2.824$, $p = 0.0135$) compared with control saline treatment. Data are expressed as individual values and as mean ± SEM. *$p < 0.05$; **$p < 0.01$, ***$p < 0.001$.

(AAV8-pEF1a-DIO-FLPo-WPRE-hGHpA) under the control of Cre into the DVC of *Vgat^{Cre}* mice, allowing us to express a second recombinase only in GABA^{DVC} cells. After recovery from surgery, a retrograde AAV encoding for a FlpO-dependent hM3Dq-mCherry (AAVrg-hSyn-fDIO-hM3D(Gq)-mCherry-WPREpA) was bilaterally injected into the ARC to retrogradely deliver hM3Dq in a FlpO-dependent manner to GABA^{DVC} cells. Therefore, hM3Dq was expressed only in GABA^{DVC} cells projecting to the ARC in *Vgat^{Cre}* mice (GABA^{DVC} → ARC:hM3Dq mice) (Figure 4A).

Corroborating the optogenetic data presented above, the selective chemogenetic stimulation of the GABA^{DVC} → ARC circuit significantly reduced acute food intake (Figures 4B and 4C) in *ad libitum*-fed mice compared with saline. GABA^{DVC} → ARC circuit activation did not alter overall locomotor activity (Figures 4D and 4E), respiratory echange ratio (RER) (Figures 4F and 4G), or heat production compared with saline treatment (Figures 4H and 4I). GABA^{DVC} → ARC neuron activation also reduced food intake in refed mice after overnight fasting (Figures 4J and 4K). Fasting is associated with a rise in systemic levels of ghrelin,[57] which acts as a pre-prandial effector stimulating AgRP/NPY neurons to initiate a feeding response.[58,59] To examine whether GABA^{DVC} → ARC neuron activation is sufficient to dampen a hunger cue, GABA^{DVC} → ARC:hM3Dq mice were pretreated with CNO prior to ghrelin. Activation of GABA^{DVC} → ARC neurons with CNO prevented the feeding induced by an orexigenic dose of ghrelin (Figures 4L and 4M). We next performed an analysis of the microstructure of the feeding event during the anorectic episode produced by CNO.[60–62] GABA^{DVC} → ARC neuron activation reduced meal size (Figure 4N) and significantly reduced the number of meal events in the earliest interval compared with saline treatment (Figures 4O and 4P). Taken

together, these findings indicate that $GABA^{DVC}$ → ARC neuron activation is sufficient to blunt fasting and ghrelin-induced hunger and significantly reduces food intake.

DISCUSSION

Here, we identify a critical new brain circuit modulating food intake and body weight. We focused on the DVC because it is a brain region positioned to receive and integrate energy-related information from the periphery and relay it within the CNS to promote energy homeostasis. However, the DVC is neurochemically heterogeneous and key neurons within it that perform this function have not been fully defined. Using a multi-methodological approach, we identify $GABA^{DVC}$ neurons as sufficient to control feeding behavior and body weight in mice.

Recent efforts to decode the function of specific chemically defined neurons within the DVC have revealed that distinct subpopulations of glutamatergic cells play a role in energy homeostasis.[4,24] However, DVC inhibitory GABA-releasing neurons have not been studied in detail, and this is necessary to clarify the role of both excitatory and inhibitory DVC signals in the regulation of energy balance.[6] Here, we provide a detailed characterization of the effect of $GABA^{DVC}$ in the regulation of energy homeostasis and body weight. A recent report provided evidence that the obesity medication liraglutide engages $GABA^{NTS}$ neurons to reduce food intake in rats, providing a rationale that activating $GABA^{NTS}$ cells may have translational relevance for the treatment of human obesity.[11]

The NTS subregion of the DVC is involved in satiety and satiation, and refeeding induces a strong neuronal activation in this region.[33] We discovered that refeeding significantly activates a subset of $GABA^{NTS}$ cells. Further, we found that chemogenetic activation of $GABA^{DVC}$ neurons reduced food intake and body weight, whereas chemogenetic inhibition of $GABA^{DVC}$ neurons stimulated feeding in satiated mice in the light cycle. However, when $GABA^{DVC}$ neurons were inhibited in mice entering the dark cycle, food intake was not increased. The likely explanation for this result is methodological. A stimulation of food intake is easier to detect during the circadian phase when mice naturally eat very little (the light cycle). In contrast, mice consume a large bout of food at the onset of the dark cycle, and this likely obscures or prevents the effect of $GABA^{DVC}$ neuron inhibition to produce the same behavior. Specifically, the natural dark cycle feeding bout produces a gut-brain feedback mechanism that activates neurons within the DVC, including $GABA^{DVC}$ neurons, thereby likely diminishing or overriding the effect of CNO treatment. Further experimentation is required to characterize this effect. Taken together, we observed that the manipulation of $GABA^{DVC}$ neuron activity produced consistent changes in feeding behavior using four different approaches, illustrating that these neurons are a controller of energy balance.

Our data illustrates that $GABA^{DVC}$ neurons project widely within the brain, with particularly dense local DVC terminals and innervation of the hypothalamus. Several studies indicate that there is coordination between the NTS and the hypothalamus to orchestrate the meal event.[14,26–28,63] Other reports reveal an indirect inhibitory input to the ARC AgRP neurons.[9] However, whether the ARC receives direct inhibitory control from the DVC is not known and was examined here.

Given that GABA is an inhibitory neurotransmitter, we hypothesized that $GABA^{DVC}$ neurons either directly or indirectly inhibit appetite-stimulating neurons. We focused on $AgRP/NPY^{ARC}$ neurons because of the dense $GABA^{DVC}$ innervation that we found and the potent orexigenic properties of AgRP/NPY neurons.[40,41,64] Specifically, $AgRP/NPY^{ARC}$ cells are poised to orchestrate the integration of homeostatic, reward, and sensory cues as well as learning and conditioned behaviors.[40,41,53,65–72] We demonstrated that $GABA^{DVC}$ terminals in the ARC release GABA and that this is synchronized with suppression of the action potential propagation on postsynaptic NPY^{ARC} cells. The timescale of $GABA_AR$ openings after optical stimulation in a membrane patch placed over a $GABA^{DVC}$ terminal suggests the release of GABA from this terminal rather than from another inhibitory neuron. Postsynaptic inhibition of NPY^{ARC} cells could potentially occur through the co-release of glycine by $GABA^{DVC}$ terminals; however, the difference of more than two orders of magnitude between the gabazine IC50 values at $GABA_AR$ (349 nM)[73] and glycine receptor (GlyR) (178.1 μM)[74] makes our working concentration of gabazine (5 μM) highly effective at $GABA_ARs$ but not at GlyRs. Therefore, the close to 100% suppression of inhibitory conductance by gabazine (Figure 2M) strongly suggests that the effects we observed are due to release of GABA rather than glycine.

The number of NPY^{ARC} neurons responding to $GABA^{DVC}$ terminal stimulation is in line with the 20% of ARC responders to NTS innervation published previously.[14] However, despite the relatively small number of NPY^{ARC} neurons inhibited by $GABA^{DVC}$ terminals, a detailed analysis of feeding behavior revealed that activation of the $GABA^{DVC}$ → NPY^{ARC} pathway specifically reduces food intake and does not induce negative valence, aversion, or anxiety. *In vivo*, indirect $GABA^{DVC}$ inputs to NPY^{ARC} neurons may also contribute to the suppression of NPY^{ARC} neuron activity and feeding behavior.

Obesity is an international health concern that is primarily the consequence of consuming more calories than the body requires. Defining the mechanisms governing hunger and food intake is therefore of paramount importance. Here, we identify a new player that controls appetite and body weight, $GABA^{DVC}$ neurons. Specifically, we show that DVC GABA-releasing neurons are active following meal ingestion and reduce food intake and body weight without causing aversion or anxiety. We demonstrate that $GABA^{DVC}$ cells directly activate $GABA_ARs$ on the surface of hunger-associated NPY^{ARC} cells, which inhibits neuron activity. These studies reveal for the first time the effect of $GABA^{DVC}$ neurons on feeding and body weight and identify a fast inhibitory circuit between the NTS and the ARC in the control of food intake. These results thereby provide significant insight into the brain circuits governing appetite and body weight, findings of relevance to the global obesity crisis.

STAR★METHODS

Detailed methods are provided in the online version of this paper and include the following:

- KEY RESOURCES TABLE
- RESOURCE AVAILABILITY
  - Lead contact

- Materials availability
- Data and code availability
- EXPERIMENTAL MODEL AND STUDY PARTICIPANT DETAILS
- METHOD DETAILS
  - RNA-Seq
  - Viral vectors
  - Stereotaxic surgeries
  - *In vivo* photo-stimulation protocol
  - Food intake and body weight studies
  - Behavioral tests
  - Immunohistochemistry and imaging
  - Electrophysiology
- QUANTIFICATION AND STATISTICAL ANALYSIS

SUPPLEMENTAL INFORMATION

Supplemental information can be found online at https://doi.org/10.1016/j.cub.2024.02.074.

ACKNOWLEDGMENTS

We gratefully acknowledge Dr F. Naneix for advice on optogenetics and editorial advice, Ms. Raffaella Chianese and staff within the University of Aberdeen Medical Research Facility, and the Microscopy Facility for their technical assistance. This work was supported by the ERC (MSCA-IF-NeuroEE-660219) to P.B.M.d.M., Wellcome Trust Institutional Strategic Support Fund (204815/Z/16/Z) to P.B.M.d.M. and L.K.H., and the Biotechnology and Biological Sciences Research Council (BB/V010557/1) to J.A.G. (BB/R01857X/1) to L.K.H and (BB/V016849/1) to L.K.H. and S.S.. G.K.C.D. is funded by a BBSRC CASE 4-year PhD studentship, co-funded by Novo Nordisk. G.S.H.Y. is funded by the UK Medical Research Council (MC_UU_00014/1). For the purpose of open access, L.K.H. has applied a Creative Commons Attribution (CC BY) license to any author accepted manuscript version arising from this submission.

AUTHOR CONTRIBUTIONS

Conceptualization, P.B.M.d.M. and L.K.H.; methodology, P.B.M.d.M., J.A.G., S.S., L.K.H., and G.S.H.Y.; software, P.B.M.d.M., J.A.G., and S.S.; validation, P.B.M.d.M.; formal analysis, P.B.M.d.M., J.A.G., G.K.C.D., G.S.H.Y., and S.S.; investigation, P.B.M.d.M., J.A.G., G.K.C.D., Y.M., and S.S.; resources, P.B.M., J.A.G., G.S.H.Y., S.S., and L.K.H.; data curation, P.B.M.d.M., J.A.G., G.K.C.D., G.S.H.Y., and S.S.; writing – original draft, P.B.M.d.M.; writing – review & editing, J.A.G., G.K.C.D., Y.M., G.S.H.Y., S.S., and L.K.H.; visualization, P.B.M.d.M. and S.S.; supervision, P.B.M.d.M. and L.K.H.; project administration, P.B.M.d.M. and L.K.H; funding acquisition, P.B.M.d.M., J.A.G., G.S.H.Y., S.S., and L.K.H.

DECLARATION OF INTERESTS

The authors declare no competing financial interests.

Received: October 17, 2023
Revised: February 5, 2024
Accepted: February 29, 2024
Published: March 21, 2024

REFERENCES

1. Andermann, M.L., and Lowell, B.B. (2017). Toward a Wiring Diagram Understanding of Appetite Control. Neuron *95*, 757–778.
2. Campos, A., Port, J.D., and Acosta, A. (2022). Integrative Hedonic and Homeostatic Food Intake Regulation by the Central Nervous System: Insights from Neuroimaging. Preprint at MDPI.
3. Hyun, U., and Sohn, J.W. (2022). Autonomic control of energy balance and glucose homeostasis. Exp. Mol. Med. *54*, 370–376.
4. Dowsett, G.K.C., Lam, B.Y.H., Tadross, J.A., Cimino, I., Rimmington, D., Coll, A.P., Polex-Wolf, J., Knudsen, L.B., Pyke, C., and Yeo, G.S.H. (2021). A survey of the mouse hindbrain in the fed and fasted states using single-nucleus RNA sequencing. Mol. Metab. *53*, 101240.
5. Grill, H.J., and Hayes, M.R. (2012). Hindbrain Neurons as an Essential Hub in the Neuroanatomically Distributed Control of Energy Balance. Cell Metab. *16*, 296–309.
6. Cheng, W., Gordian, D., Ludwig, M.Q., Pers, T.H., Seeley, R.J., and Myers, M.G. (2022). Hindbrain circuits in the control of eating behaviour and energy balance. Nat. Metab. *4*, 826–835.
7. Cheng, W., Ndoka, E., Hutch, C., Roelofs, K., MacKinnon, A., Khoury, B., Magrisso, J., Kim, K.S., Rhodes, C.J., Olson, D.P., et al. (2020). Leptin receptor-expressing nucleus tractus solitarius neurons suppress food intake independently of GLP1 in mice. JCI Insight *5*, e134359.
8. Hayes, M.R., Skibicka, K.P., Leichner, T.M., Guarnieri, D.J., DiLeone, R.J., Bence, K.K., and Grill, H.J. (2010). Endogenous Leptin Signaling in the Caudal Nucleus Tractus Solitarius and Area Postrema Is Required for Energy Balance Regulation. Cell Metab. *11*, 77–83.
9. Cheng, W., Gonzalez, I., Pan, W., Tsang, A.H., Adams, J., Ndoka, E., Gordian, D., Khoury, B., Roelofs, K., Evers, S.S., et al. (2020). Calcitonin Receptor Neurons in the Mouse Nucleus Tractus Solitarius Control Energy Balance via the Non-aversive Suppression of Feeding. Cell Metab. *31*, 301–312.e5.
10. Alhadeff, A.L., Mergler, B.D., Zimmer, D.J., Turner, C.A., Reiner, D.J., Schmidt, H.D., Grill, H.J., and Hayes, M.R. (2017). Endogenous Glucagon-like Peptide-1 Receptor Signaling in the Nucleus Tractus Solitarius is Required for Food Intake Control. Neuropsychopharmacology *42*, 1471–1479.
11. Fortin, S.M., Lipsky, R.K., Lhamo, R., Chen, J., Kim, E., Borner, T., Schmidt, H.D., and Hayes, M.R. (2020). GABA neurons in the nucleus tractus solitarius express GLP-1 receptors and mediate anorectic effects of liraglutide in rats HHS Public Access. Sci. Transl. Med. *12*, 109–114.
12. Tang-Christensen, M., Larsen, P.J., Göke, R., Fink-Jensen, A., Jessop, D.S., Møller, M., and Sheikh, S.P. (1996). Central administration of GLP-1-(7-36) amide inhibits food and water intake in rats. Am. J. Physiol. Regul. Integr. Comp. Physiol. *271*, R848–R856.
13. Zheng, H., Stornetta, R.L., Agassandian, K., and Rinaman, L. (2015). Glutamatergic phenotype of glucagon-like peptide 1 neurons in the caudal nucleus of the solitary tract in rats. Brain Struct. Funct. *220*, 3011–3022.
14. Aklan, I., Sayar Atasoy, N., Yavuz, Y., Ates, T., Coban, I., Koksalar, F., Filiz, G., Topcu, I.C., Oncul, M., Dilsiz, P., et al. (2020). NTS Catecholamine Neurons Mediate Hypoglycemic Hunger via Medial Hypothalamic Feeding Pathways. Cell Metab. *31*, 313–326.e5.
15. Chen, J., Cheng, M., Wang, L., Zhang, L., Xu, D., Cao, P., Wang, F., Herzog, H., Song, S., and Zhan, C. (2020). A Vagal-NTS Neural Pathway that Stimulates Feeding. Curr. Biol. *30*, 3986–3998.e5.
16. Rinaman, L., Baker, E.A., Hoffman, G.E., Stricker, E.M., and Verbalis, J.G. (1998). Medullary c-Fos activation in rats after ingestion of a satiating meal. Am. J. Physiol. *275*, R262–R268.
17. D'Agostino, G., Lyons, D.J., Cristiano, C., Burke, L.K., Madara, J.C., Campbell, J.N., Garcia, A.P., Land, B.B., Lowell, B.B., Dileone, R.J., et al. (2016). Appetite controlled by a cholecystokinin nucleus of the solitary tract to hypothalamus neurocircuit. eLife *5*.
18. Roman, C.W., Derkach, V.A., and Palmiter, R.D. (2016). Genetically and functionally defined NTS to PBN brain circuits mediating anorexia. Nat. Commun. *7*, 11905.
19. Ellacott, K.L.J., Lawrence, C.B., Rothwell, N.J., and Luckman, S.M. (2002). PRL-Releasing Peptide Interacts with Leptin to Reduce Food Intake and Body Weight. Endocrinology *143*, 368–374.
20. Chen, C.T., Dun, S.L., Dun, N.J., and Chang, J.K. (1999). Prolactin-releasing peptide-immunoreactivity in A1 and A2 noradrenergic neurons of the rat medulla. Brain Res. *822*, 276–279.

21. Bechtold, D.A., and Luckman, S.M. (2006). Prolactin-Releasing Peptide Mediates Cholecystokinin-Induced Satiety in Mice. Endocrinology *147*, 4723–4729.

22. Zhan, C., Zhou, J., Feng, Q., Zhang, J.E., Lin, S., Bao, J., Wu, P., and Luo, M. (2013). Acute and long-term suppression of feeding behavior by POMC neurons in the brainstem and hypothalamus, respectively. J. Neurosci. *33*, 3624–3632.

23. Georgescu, T., Lyons, D., Doslikova, B., Garcia, A.P., Marston, O., Burke, L.K., Chianese, R., Lam, B.Y.H., Yeo, G.S.H., Rochford, J.J., et al. (2020). Neurochemical Characterization of Brainstem Pro-Opiomelanocortin Cells. Endocrinology *161*.

24. Ludwig, M.Q., Cheng, W., Gordian, D., Lee, J., Paulsen, S.J., Hansen, S.N., Egerod, K.L., Barkholt, P., Rhodes, C.J., Secher, A., et al. (2021). A genetic map of the mouse dorsal vagal complex and its role in obesity. Nat. Metab. *3*, 530–545.

25. Shi, M.Y., Ding, L.F., Guo, Y.H., Cheng, Y.X., Bi, G.Q., and Lau, P.M. (2021). Long-range GABAergic projections from the nucleus of the solitary tract. Mol. Brain *14*, 38.

26. Blevins, J.E., Schwartz, M.W., and Baskin, D.G. (2004). Evidence that paraventricular nucleus oxytocin neurons link hypothalamic leptin action to caudal brain stem nuclei controlling meal size. Am. J. Physiol. Regul. Integr. Comp. Physiol. *287*, R87–R96.

27. Liu, J., Conde, K., Zhang, P., Lilascharoen, V., Xu, Z., Lim, B.K., Seeley, R.J., Zhu, J.J., Scott, M.M., and Pang, Z.P. (2017). Enhanced AMPA receptor trafficking mediates the anorexigenic effect of endogenous glucagon like peptide-1 in the paraventricular hypothalamus. Neuron *96*, 897–909.e5.

28. Tsang, A.H., Nuzzaci, D., Darwish, T., Samudrala, H., and Blouet, C. (2020). Nutrient sensing in the nucleus of the solitary tract mediates nonaversive suppression of feeding via inhibition of AgRP neurons. Mol. Metab. *42*, 101070.

29. Betley, J.N., Cao, Z.F.H., Ritola, K.D., and Sternson, S.M. (2013). Parallel, Redundant Circuit Organization for Homeostatic Control of Feeding Behavior. Cell *155*, 1337–1350.

30. Heisler, L.K., and Lam, D.D. (2017). An appetite for life: brain regulation of hunger and satiety. Curr. Opin. Pharmacol. *37*, 100–106.

31. Heisler, L.K., Jobst, E.E., Sutton, G.M., Zhou, L., Borok, E., Thornton-Jones, Z., Liu, H.Y., Zigman, J.M., Balthasar, N., Kishi, T., et al. (2006). Serotonin Reciprocally Regulates Melanocortin Neurons to Modulate Food Intake. Neuron *51*, 239–249.

32. Hahn, T.M., Breininger, J.F., Baskin, D.G., and Schwartz, M.W. (1998). Coexpression of Agrp and NPY in fasting-activated hypothalamic neurons. Nat. Neurosci. *1*, 271–272.

33. Olson, B.R., Freilino, M., Hoffman, G.E., Stricker, E.M., Sved, A.F., and Verbalis, J.G. (1993). C-Fos expression in rat brain and brainstem nuclei in response to treatments that alter food intake and gastric motility. Mol. Cell. Neurosci. *4*, 93–106.

34. Alexander, G.M., Rogan, S.C., Abbas, A.I., Armbruster, B.N., Pei, Y., Allen, J.A., Nonneman, R.J., Hartmann, J., Moy, S.S., Nicolelis, M.A., et al. (2009). Remote control of neuronal activity in transgenic mice expressing evolved G protein-coupled receptors. Neuron *63*, 27–39.

35. Jensen, C.B., Pyke, C., Rasch, M.G., Dahl, A.B., Knudsen, L.B., and Secher, A. (2018). Characterization of the Glucagonlike Peptide-1 Receptor in Male Mouse Brain Using a Novel Antibody and In Situ Hybridization. Endocrinology *159*, 665–675.

36. Cork, S.C., Richards, J.E., Holt, M.K., Gribble, F.M., Reimann, F., and Trapp, S. (2015). Distribution and characterisation of Glucagon-like peptide-1 receptor expressing cells in the mouse brain. Mol. Metab. *4*, 718–731.

37. Graham, D.L., Durai, H.H., Trammell, T.S., Noble, B.L., Mortlock, D.P., Galli, A., and Stanwood, G.D. (2020). A novel mouse model of glucagon-like peptide-1 receptor expression: A look at the brain. J. Comp. Neurol. *528*, 2445–2470.

38. Petreanu, L., Huber, D., Sobczyk, A., and Svoboda, K. (2007). Channelrhodopsin-2-assisted circuit mapping of long-range callosal projections. Nat. Neurosci. *10*, 663–668.

39. González, J.A., Iordanidou, P., Strom, M., Adamantidis, A., and Burdakov, D. (2016). Awake dynamics and brain-wide direct inputs of hypothalamic MCH and orexin networks. Nat. Commun. *7*, 11395.

40. Krashes, M.J., Koda, S., Ye, C.P., Rogan, S.C., Adams, A.C., Cusher, D.S., Maratos-Flier, E., Roth, B.L., and Lowell, B.B. (2011). Rapid, reversible activation of AgRP neurons drives feeding behavior in mice. J. Clin. Invest. *121*, 1424–1428.

41. Aponte, Y., Atasoy, D., and Sternson, S.M. (2011). AGRP neurons are sufficient to orchestrate feeding behavior rapidly and without training. Nat. Neurosci. *14*, 351–355.

42. Krnjević, K., and Schwartz, S. (1967). The action of γ-Aminobutyric acid on cortical neurones. Exp. Brain Res. *3*, 320–336.

43. Williams, D.L., and Schwartz, M.W. (2005). The melanocortin system as a central integrator of direct and indirect controls of food intake. Am. J. Physiol. Regul. Integr. Comp. Physiol. *289*, R2–R3.

44. Spruston, N., Jaffe, D.B., Williams, S.H., and Johnston, D. (1993). Voltage- and space-clamp errors associated with the measurement of electrotonically remote synaptic events. J. Neurophysiol. *70*, 781–802.

45. Lindroos, R., and Hellgren Kotaleski, J. (2021). Predicting complex spikes in striatal projection neurons of the direct pathway following neuromodulation by acetylcholine and dopamine. Eur. J. Neurosci. *53*, 2117–2134.

46. Sylantyev, S., Savtchenko, L.P., O'Neill, N., and Rusakov, D.A. (2020). Extracellular GABA waves regulate coincidence detection in excitatory circuits. J. Physiol. *598*, 4047–4062.

47. D'Agostino, G., and Luckman, S.M. (2022). Brainstem peptides and peptidergic neurons in the regulation of appetite. Curr. Opin. Endocr. Metab. Res. *24*, 100339.

48. Betley, J.N., Xu, S., Cao, Z.F.H., Gong, R., Magnus, C.J., Yu, Y., and Sternson, S.M. (2015). Neurons for hunger and thirst transmit a negative-valence teaching signal. Nature *521*, 180–185.

49. Berridge, K.C. (2004). Motivation concepts in behavioral neuroscience. Physiol. Behav. *81*, 179–209.

50. Stamatakis, A.M., and Stuber, G.D. (2012). Activation of lateral habenula inputs to the ventral midbrain promotes behavioral avoidance. Nat. Neurosci. *15*, 1105–1107.

51. Kim, S.Y., Adhikari, A., Lee, S.Y., Marshel, J.H., Kim, C.K., Mallory, C.S., Lo, M., Pak, S., Mattis, J., Lim, B.K., et al. (2013). Diverging neural pathways assemble a behavioural state from separable features in anxiety. Nature *496*, 219–223.

52. Li, C., Hou, Y., Zhang, J., Sui, G., Du, X., Licinio, J., Wong, M.L., and Yang, Y. (2019). AGRP neurons modulate fasting-induced anxiolytic effects. Transl. Psychiatry *9*, 111.

53. Dietrich, M.O., Zimmer, M.R., Bober, J., and Horvath, T.L. (2015). Hypothalamic Agrp Neurons Drive Stereotypic Behaviors beyond Feeding. Cell *160*, 1222–1232.

54. Heinz, D.E., Schöttle, V.A., Nemcova, P., Binder, F.P., Ebert, T., Domschke, K., and Wotjak, C.T. (2021). Exploratory drive, fear, and anxiety are dissociable and independent components in foraging mice. Transl. Psychiatry *11*, 318.

55. Fenno, L.E., Mattis, J., Ramakrishnan, C., and Deisseroth, K. (2017). A Guide to Creating and Testing New INTRSECT Constructs. Curr. Protoc. Neurosci. *80*, 4.39.1–4.39.24.

56. Fenno, L.E., Mattis, J., Ramakrishnan, C., Hyun, M., Lee, S.Y., He, M., Tucciarone, J., Selimbeyoglu, A., Berndt, A., Grosenick, L., et al. (2014). Targeting cells with single vectors using multiple-feature Boolean logic. Nat. Methods *11*, 763–772.

57. Cummings, D.E., Purnell, J.Q., Frayo, R.S., Schmidova, K., Wisse, B.E., and Weigle, D.S. (2001). A Preprandial Rise in Plasma Ghrelin Levels Suggests a Role in Meal Initiation in Humans. Diabetes *50*, 1714–1719.

58. Chen, H.Y., Trumbauer, M.E., Chen, A.S., Weingarth, D.T., Adams, J.R., Frazier, E.G., Shen, Z., Marsh, D.J., Feighner, S.D., Guan, X.M., et al.

(2004). Orexigenic Action of Peripheral Ghrelin Is Mediated by Neuropeptide Y and Agouti-Related Protein. Endocrinology *145*, 2607–2612.

59. Luquet, S., Phillips, C.T., and Palmiter, R.D. (2007). NPY/AgRP neurons are not essential for feeding responses to glucoprivation. Peptides *28*, 214–225.

60. Clifton, P.G. (2000). Meal patterning in rodents: psychopharmacological and neuroanatomical studies. Neurosci. Biobehav. Rev. *24*, 213–222.

61. Richard, C.D., Tolle, V., and Low, M.J. (2011). Meal pattern analysis in neural-specific proopiomelanocortin-deficient mice. Eur. J. Pharmacol. *660*, 131–138.

62. Zorrilla, E.P., Inoue, K., Fekete, É.M., Tabarin, A., Valdez, G.R., and Koob, G.F. (2005). Measuring meals: Structure of prandial food and water intake of rats. Am. J. Physiol. Regul. Integr. Comp. Physiol. *288*, R1450–R1467.

63. D'Agostino, G., Lyons, D., Cristiano, C., Lettieri, M., Olarte-Sanchez, C., Burke, L.K., Greenwald-Yarnell, M., Cansell, C., Doslikova, B., Georgescu, T., et al. (2018). Nucleus of the Solitary Tract Serotonin 5-HT2C Receptors Modulate Food Intake. Cell Metab. *28*, 619–630.e5.

64. Luquet, S., Perez, F.A., Hnasko, T.S., and Palmiter, R.D. (2005). NPY/AgRP neurons are essentials for feeding in adult mice but can be ablated in neonates. Science *310*, 683–685.

65. Berrios, J., Li, C., Madara, J.C., Garfield, A.S., Steger, J.S., Krashes, M.J., and Lowell, B.B. (2021). Food cue regulation of AGRP hunger neurons guides learning. Nature *595*, 695–700.

66. Deem, J.D., Faber, C.L., and Morton, G.J. (2022). AgRP neurons: Regulators of feeding, energy expenditure, and behavior. FEBS Journal *289*, 2362–2381.

67. Dietrich, M.O., Bober, J., Ferreira, J.G., Tellez, L.A., Mineur, Y.S., Souza, D.O., Gao, X.-B., Picciotto, M.R., Araújo, I., Liu, Z.-W., and Horvath, T.L. (2012). AgRP neurons regulate development of dopamine neuronal plasticity and nonfood-associated behaviors. Nat. Neurosci. *15*, 1108–1110.

68. Garau, C., Blomeley, C., and Burdakov, D. (2020). Orexin neurons and inhibitory Agrp→orexin circuits guide spatial exploration in mice. J. Physiol. *598*, 4371–4383.

69. Han, Y., Xia, G., Srisai, D., Meng, F., He, Y., Ran, Y., He, Y., Farias, M., Hoang, G., Tóth, I., et al. (2021). Deciphering an AgRP-serotoninergic neural circuit in distinct control of energy metabolism from feeding. Nat. Commun. *12*, 3525.

70. Jikomes, N., Ramesh, R.N., Mandelblat-Cerf, Y., and Andermann, M.L. (2016). Preemptive Stimulation of AgRP Neurons in Fed Mice Enables Conditioned Food Seeking under Threat. Curr. Biol. *26*, 2500–2507.

71. Krashes, M.J., Shah, B.P., Madara, J.C., Olson, D.P., Strochlic, D.E., Garfield, A.S., Vong, L., Pei, H., Watabe-Uchida, M., Uchida, N., et al. (2014). An excitatory paraventricular nucleus to AgRP neuron circuit that drives hunger. Nature *507*, 238–242.

72. Wang, C., Zhou, W., He, Y., Yang, T., Xu, P., Yang, Y., Cai, X., Wang, J., Liu, H., Yu, M., et al. (2021). AgRP neurons trigger long-term potentiation and facilitate food seeking. Transl. Psychiatry *11*, 11.

73. Bhagat, K., Singh, J.V., Pagare, P.P., Kumar, N., Sharma, A., Kaur, G., Kinarivala, N., Gandu, S., Singh, H., Sharma, S., and P.M.S.. (2021). Rational approaches for the design of various GABA modulators and their clinical progression. Mol. Divers. *25*, 551–601.

74. Li, P., and Slaughter, M. (2007). Glycine receptor subunit composition alters the action of GABA antagonists. Vis. Neurosci. *24*, 513–521.

75. Hentges, S.T., Otero-Corchon, V., Pennock, R.L., King, C.M., and Low, M.J. (2009). Proopiomelanocortin Expression in both GABA and Glutamate Neurons. J. Neurosci. *29*, 13684–13690.

76. Vong, L., Ye, C., Yang, Z., Choi, B., Chua, S., and Lowell, B.B. (2011). Leptin action on GABAergic neurons prevents obesity and reduces inhibitory tone to POMC neurons. Neuron *71*, 142–154.

77. van den Pol, A.N., Yao, Y., Fu, L.Y., Foo, K., Huang, H., Coppari, R., Lowell, B.B., and Broberger, C. (2009). Neuromedin B and gastrin-releasing peptide excite arcuate nucleus neuropeptide Y neurons in a novel transgenic mouse expressing strong Renilla green fluorescent protein in NPY neurons. J. Neurosci. *29*, 4622–4639.

78. Madisen, L., Zwingman, T.A., Sunkin, S.M., Oh, S.W., Zariwala, H.A., Gu, H., Ng, L.L., Palmiter, R.D., Hawrylycz, M.J., Jones, A.R., et al. (2010). A robust and high-throughput Cre reporting and characterization system for the whole mouse brain. Nat. Neurosci. *13*, 133–140.

79. Hao, Y., Hao, S., Andersen-Nissen, E., Mauck, W.M., Zheng, S., Butler, A., Lee, M.J., Wilk, A.J., Darby, C., Zager, M., et al. (2021). Integrated analysis of multimodal single-cell data. Cell *184*, 3573–3587.e29.

80. Zingg, B., Chou, X.L., Zhang, Z.G., Mesik, L., Liang, F., Tao, H.W., and Zhang, L.I. (2017). AAV-Mediated Anterograde Transsynaptic Tagging: Mapping Corticocollicular Input-Defined Neural Pathways for Defense Behaviors. Neuron *93*, 33–47.

81. Wagner, S., Brierley, D.I., Leeson-Payne, A., Jiang, W., Chianese, R., Lam, B.Y.H., Dowsett, G.K.C., Cristiano, C., Lyons, D., Reimann, F., et al. (2023). Obesity medication lorcaserin activates brainstem GLP-1 neurons to reduce food intake and augments GLP-1 receptor agonist induced appetite suppression. Mol. Metab. *68*, 101665.

82. Lin, J.Y., Lin, M.Z., Steinbach, P., and Tsien, R.Y. (2009). Characterization of Engineered Channelrhodopsin Variants with Improved Properties and Kinetics. Biophys. J. *96*, 1803–1814.

83. Yavari, A., Stocker, C.J., Ghaffari, S., Wargent, E.T., Steeples, V., Czibik, G., Pinter, K., Bellahcene, M., Woods, A., Martínez de Morentin, P.B., et al. (2016). Chronic Activation of gamma2 AMPK Induces Obesity and Reduces beta Cell Function. Cell Metab. *23*, 821–836.

STAR★METHODS

KEY RESOURCES TABLE

REAGENT or RESOURCE	SOURCE	IDENTIFIER
Antibodies		
Rabbit ani-c-Fos antibody	Cell Signaling	Cat#2250; RRID:AB_2247211
Rabbit anti-c-Fos antibody	Merk	Cat#ABE457; RRID:AB_2631318
Goat anti-mCherry antibody	Scigen	Cat#AB0040-200; RRID:AB_2333093
Rabbit anti-hrGFP antibody	Agilent	Cat#240141; RRID:AB_10596971
Rabitt anti-POMC antibody	Phoenix Pharmaceuticals	Cat#H-029-30; RRID:AB_2307442
Rabbit anti-RFP antibody	Rockland Immunochemicals	Cat#600-401-379; RRID:AB_2209751
Donkey anti-rabbit AlexaFluor 488 antibody	Invitrogen	Cat#A78948; RRID:AB_2921070
Donkey anti-chicken AlexaFluor 488 antibody	Jackson immunoresearch	Cat# 703-545-155; RRID:AB_2340375
Donkey anti-rabbit AlexaFluor 594 antibody	Invitrogen	Cat#A-21207; RRID:AB_141637
Donkey anti-goat AlexaFluor 594 antibody	Invitrogen	Cat#A-11058; RRID:AB_2534105
Donkey anti-goat Biotin-SP	Jackson immunoresearch	Cat# 711-065-152; RRID:AB_2340593
Bacterial and virus strains		
AAV8-hSyn-DIO-hM3D(Gq)-mCherry	Addgene	Cat#44361-AAV8; RRID:Addgene_44361
AAV8-hSyn-DIO-hM4D(Gi)-mCherry	Addgene	Cat#44362-AAV8; RRID:Addgene_44362
AAVrg-hSyn-fDIO-hM3D (Gq)-mCherry-WPREpA	Addgene	Cat#154868-AAVrg; RRID:Addgene_154868
AAV8-hSyn-DIO-mCherry	Addgene	Cat#50459-AAV8; RRID:Addgene_50459
AAV8-pEF1a-DIO-FLPo-WPRE-hGHpA	Addgene	Cat#87306-AAV8; RRID:Addgene_87306
AAV2-EF1a-DIO-ChR2 (E123T/T159C)-mCherry	University of North Carolina Vector Core	N/A
AAV2-EF1a-DIO-ChR2 (E123T/T159C)-YFP	University of North Carolina Vector Core	N/A
Chemicals, peptides, and recombinant proteins		
Clozapine-N-Oxide	Tocris	Cat#4936
Liraglutide	Tocris	Cat#6517
Ghrelin	Tocris	Cat#1463
Gabazine	Tocris	Cat#1262
GABA	Tocris	Cat#0344
CGP-55845	Tocris	Cat#1248
MDL-72222	Tocris	Cat#0640
QX-314	Tocris	Cat#2313
ImmPACT® DAB Substrate Kit, Peroxidase	Vector Laboratories	Cat#SK-4105
VECTASTAIN® ABC-HRP Kit, Peroxidase (Standard)	Vector Laboratories	Cat#PK-4000
Experimental models: Organisms/strains		
Mouse: Slc32a1tm2(cre)Lowl	The Jackson Laboratory	Cat#016962; RRID:IMSR_JAX:016962
Mouse: B6.FVB-Tg(Npy-hrGFP)1Lowl/J	The Jackson Laboratory	Cat#006417; RRID:IMSR_JAX:006417
Mouse: B6.Cg-Gt(ROSA)26Sortm9 (CAG-tdTomato)Hze/J	The Jackson Laboratory	Cat#007909; RRID:IMSR_JAX:007909
Mouse: Tg(Pomc-DsRed)18Low	Prof. Malcom Low (University of Michigan)	Hentges et al.[75]
Software and algorithms		
Prism 10	GraphPad Software	https://www.graphpad.com/ RRID:SCR_002798
ImageJ	NIH	https://imagej.net/ij/RRID:SCR_003070
Affinity Designer 2	Serif	https://affinity.serif.com/

(Continued on next page)

Continued		
REAGENT or RESOURCE	SOURCE	IDENTIFIER
Arduino IDE	Arduino	https://www.arduino.cc/ RRID:SCR_024884
ANYmaze	Stoelting	https://www.any-maze.com/ RRID:SCR_014289
Phenomaster	TSE-Systems	https://www.tse-systems.com/
Axiovision	Zen	https://www.micro-shop.zeiss.com/ RRID:SCR_002677
RNAseq data	Data are available from the NCBI Gene Expression Omnibus	Accession GSE168737
Other		
473-nm laser	Laserglow	Cat#LRS-0473
Optic fibers 200um, 0.39NA	Thorlabs	Cat#CFMLC
Patch cables	Thorlabs	Cat#M83L1
Microinjector	Narishige	Cat#IM-11-2

RESOURCE AVAILABILITY

Lead contact

Further information and requests for resources and reagents should be directed to and will be fulfilled by the lead contact, Pablo B Martinez de Morentin (p.demorentin@leeds.ac.uk).

Materials availability

This study did not generate new unique reagents.

Data and code availability

- All data reported in this paper will be shared by the lead contact upon request.
- This paper des not report original code.
- Any additional information required to reanalyze the data reported in this paper is available from the lead contact upon request.

EXPERIMENTAL MODEL AND STUDY PARTICIPANT DETAILS

Vgat-ires-Cre (Slc32a1tm2(cre)Lowl; #016962, Vong et al.[76]), *Npy-hrGFP* (B6.FVB-Tg(Npy-hrGFP)1Lowl/J; #006417, van den Pol et al.[77]) and *Rosa26tdTomato-LoxP* (B6.Cg-Gt(ROSA)26Sortm9(CAG-tdTomato)Hze/J, #007909, Madisen et al.[78]) mice were obtained from The Jackson Laboratory (Bar Harbor, USA) and bred on a C57Bl/6J background. POMC-dsRed (Tg(Pomc-DsRed)18Low, Hentges et al.[75]) mice were a generous gift from Prof Malcom Low, University of Michigan. Male and female mice were used. Mice were fed standard laboratory chow (Standard CRM (P) 801722, Special diets, UK) and provided with water *ad libitum*, unless otherwise stated. Mice were housed in a 12-hr light:dark cycle (7:00 am-7.00 pm) in environmental controlled conditions (20-22°C and 40-60% relative humidity). All experimental procedures were performed in accordance with the UK Animal (Scientific Procedures) Act 1986 and local Ethical Review Board approval.

METHOD DETAILS

RNA-Seq

Single nucleus RNA-sequencing (Nuc-Seq) data from the mouse hindbrain in the fed and fasted state was taken from Dowsett et.al.[4] Neuronal nuclei expressing at least 1 UMI count for *Slc32a1* were identified as GABAergic neurons, subsetted and reclustered using Seurat package version 4.3.[79] Marker genes for each cluster were calculated using Wilcoxon's rank-sum test. Each cluster was named with 2 marker genes that were expressed in >60% of the cluster, <30% of the rest of the data and had an average log fold change >0.5. If no genes fit these criteria, then the two genes with the lowest p-values were used. Differential gene expression analysis between *ad libitum* fed and overnight fasted cells was performed using the Wilcoxon's rank sum test. Feature plots were drawn using the Seurat package and ggplot2.

Viral vectors

Cre-dependent viral vectors purchased from Addgene include AAV8-hSyn-DIO-hM3D(Gq)-mCherry (1.83×10^{12} gc/ml) and AAV8-hSyn-DIO-hM4D(Gi)-mCherry (1.7×10^{12} gc/ml) were a gift from Prof Bryan Roth (Addgene plasmid # 44361, Krashes et al.[40]),

CellPress
OPEN ACCESS

AAVrg-hSyn-fDIO-hM3D(Gq)-mCherry-WPREpA ($1.8x10^{12}$ gc/ml) was a gift from Prof Ulrik Gether (Addgene plasmid # 154868), AAV8-hSyn-DIO-mCherry ($3.6x10^{12}$ gc/ml) was a gift from Prof Bryan Roth (Addgene plasmid # 50459), AAV8-pEF1a-DIO-FLPo-WPRE-hGHpA ($2x10^{12}$ gc/ml) was a gift from Li Zhang (Addgene plasmid # 87306, Zingg et al.[80]). AAV2-EF1a-DIO-ChR2(E123T/T159C)-mCherry and AAV2-EF1a-DIO-ChR2(E123T/T159C)-YFP ($7.3x10^{12}$ vp/ml) were a gift from Prof Karl Deisseroth and were obtained from University of North Carolina Vector Core (Chapel Hill, NC, USA). All viral particles were delivered into nuclei-specific regions through stereotaxic injections.

Stereotaxic surgeries

For viral delivery into the DVC, stereotaxic surgery was adapted from previous studies.[17] Briefly, 12–20 week-old mice were anaesthetized with isoflurane, the back region of the neck was shaved, and mice were placed in a stereotaxic instrument (David Kopf instruments, CA, USA) with a face mask (World Precision Instruments, FL, USA). The head was inclined ~70 degrees forward and a longitudinal incision was made at the level of the C1 and neck muscles were retracted to expose the atlanto-occipital membrane. This was carefully dissected allowing access to the dorsal brainstem and visualization of the obex. Using a pulled glass capillary (40μm tip diameter) (G1, Narishige, UK) and a pneumatic microinjector (IM-11-2, Narishige, UK), 200-300 nl of viral preparation was bilaterally injected into the NTS component of the DVC (obex: AP:0.25 mm AP, L:± 0.25 mm and DV:-0.25mm) at a flow of 50nl/minute. The capillary was left in the injection place for 5 minutes to allow diffusion and it was removed slowly to avoid dispersion to neighbor brainstem regions. Viral delivery into the ARC was performed as previously described[81] at coordinates bregma: AP:1.58 mm AP, L:±0.2mm and DV:5.90 mm. For optical fiber cannula placement, mice were allowed 4 weeks recovery form the DVC surgery and a 200 μm core diameter, 0.39NA (CFMLC, Thorlabs, UK) optical fiber implant was placed in the third ventricle above the ARC. Mice were left 3 weeks to allow surgical recovery and maximal viral expression. Postmortem analysis of injection site, viral expression and cannula placement were used as inclusion criteria for data analysis.

In vivo photo-stimulation protocol

Optical fiber implants were attached to optogenetics patch cables (M83L1, Thorlabs, UK) connected to a rotary joint (Doric lenses) coupled to a 473-nm laser (Laserglow, Toronto, Canada) controlled via TTL-USB interface with Arduino board. For feeding experiments, the stimulation protocol was 1s followed by 4s break with 10ms light pulses with a frequency of 30Hz. For behavioral experiments, the stimulation protocol was 1s followed by 0.5s break with 10ms light pulses with a frequency of 30Hz. We used 15mW of laser power to achieve an irradiance of 5-10mW/mm^2 (PM100D, Thorlabs) in the target area following https://web.stanford.edu/group/dlab/cgi-bin/graph/chart.php above ChR2 threshold activation.[82]

Food intake and body weight studies

For food intake, body weight and metabolic parameters measurements, mice were single housed and habituated in indirect calorimetry system cages for one week (Phenomaster, TSE Systems, Germany). For acute *ad libitum* studies, access to food was removed in fed mice 2 hours before entering the dark cycle and CNO 1 mg/kg was i.p. administered 30 minutes before the dark cycle onset, at which time food was provided. For re-feeding studies, 12 hour dark cycle-food deprived mice were i.p. injected with CNO at the beginning of the light cycle and 30 minutes after food was provided. For daily CNO treatment studies, mice were i.p. injected twice a day (am and pm) for 5 days with CNO following 5 days with saline. For studies with liraglutide (0.004mg/kg; Tocris, UK) and ghrelin (0.5mg/kg; Tocris, UK), drugs were administered i.p at the same time as CNO or saline.

Behavioral tests

For valence studies, mice were assessed in an adapted real-time place preference task consisting in an open field arena with two connected identical chambers (30x25cm),[17,50,51] one of them paired with optogenetic stimulation where mice were allowed free movement for 20 minutes. For food interaction studies, mice were placed in an open arena (50x50cm) for 10 minutes and an object (novel, nutritional known (chow), and a novel palatable) was randomly allocated in previously defined regions. For anxiety tests, mice were placed in an open arena (50x50cm) with virtual delimited central and peripheral regions and allowed free movement for 10 minutes with and without stimulation in different days. For anxiety and fear assessment, mice were placed in an elevated zero maze (diameter 50cm, elevation 70 cm) with 2 hidden and 2 exposed zones and allowed free movement between zones for 10 minutes. Tests were performed for each animal with and without stimulation in different days. Time and locomotor parameters for each task and zone were recorded using Any-Maze software (Stoelting, IL, USA).

Immunohistochemistry and imaging

All mice were injected with a terminal dose of anesthesia and transcardially perfused with phosphate-buffered saline (PBS) followed by 10% neutral buffered formalin. Brains were dissected, post-fixed 12 hours in formalin at 4°C, cryoprotected 48 hours with 30% sucrose 4°C and coronally sectioned in 5 series at 25 μm using a freezing microtome (8000, Bright Instruments, UK). Sections were kept in protective anti-freeze solution at 4°C until they were processed for immunohistochemistry as previously described.[83] Briefly, NTS sections were washed with PBS-0.2% Tween20 30 minutes and then PBS (3x10 minutes), blocked with 1%BSA/5%DS/0.25% Triton X-100 1 hour at room temperature and incubated with primary antibody in blocking solution for 16 hours at room temperature for fluorescence detection. Primary antibodies used include anti-c-Fos (1:2500, 2250, CST, USA), anti-mCherry (1:2000, AB0040-200, Scigen, PT), anti-RFP (1:1000, 600-401-379, Rockland Immunochemicals, USA), anti-POMC (1:3000, H-029-30, Phoenix

Pharmaceuticals, USA), anti-hrGFP (1:2000, 240141, Agilent, USA). The next day, sections were washed with PBS-Tween and PBS and incubated 1 hour with appropriate secondary antibodies in blocking solution (1:500, AlexaFluor594, AlexaFluor488, Invitrogen, UK) at room temperature. For c-Fos expression quantification in fast vs refed study, chromogenic staining with 3,3′-diaminobenzidine (DAB) reagent was performed. Briefly, endogenous peroxidase was blocked for 20 minutes using 1% hydroxide peroxide prior to overnight incubation with anti-c-Fos (1:5000, ABE457, Merck, UK). The next day, tissue was incubated with biotin-SP donkey anti-rabbit (1:500, 711-065-125, Jackson Immunoresearch, USA) for 1 hour at room temperature. Avidin/Biotin peroxidase system (PK-6100, VectorLabs, USA) and DAB developing kits were used following the manufacturer instructions to generate the chromogenic signal.

Images were acquired using Axioskope2 microscope and Axiovision software (Zeiss, Germany). For analysis, images were converted to 8-bit, pseudo-recolored and counted manually using ImageJ (Fiji) software.

Electrophysiology

CRACM study

CRACM experiments were performed as previously described.[39] Six *Vgat*^{Cre}::*Npy*^{hrGFP} mice and four *Vgat*^{Cre}::*Pomc*^{dsRed} mice were bilaterally injected with AAV2-EF1a-DIO-ChR2(E123T/T159C)-mCherry or AAV2-EF1a-DIO-ChR2(E123T/T159C)-YFP, respectively, into the DVC aimed at the NTS. Male and female mice were aged between 5 and 7 months at the time of the electrophysiology experiments. Coronal hypothalamic brain sections 180-μm thick were prepared from these mice at least 8 days after virus injections and were placed in a bath solution consisting of (in mM) 125 NaCl, 2.5 KCl, 1.2 NaH_2PO_4, 21 $NaHCO_3$, 1 glucose, 2 $MgCl_2$, 2 $CaCl_2$. hrGFP or DsRed expressing cells in the ARC were identified using an upright microscope (Scientifica S-Scope-II) equipped with the appropriate fluorescence filters. Whole-cell recordings from these cells were obtained with glass pipettes (World Precision Instruments 1B150F-4) filled with a solution containing (in mM) 120 K-gluconate, 10 HEPES, 10 KCl, 1 EGTA, 2 MgCl2, 4K2ATP, and 1 Na2ATP, tip resistance 3-7 MOhm. Data was acquired using Axon Instruments hardware (MultiClamp 700B, Digidata 1550). To test for GABA inputs to NPY^{ARC} cells, the membrane potential in these cells was clamped at increasing levels of voltage (from −100 to −10 mV in 10-mV increments), while ChR2-expressing terminals were stimulated by a single light pulse (CoolLED pE-4000) to induce post-synaptic currents. Liquid junction potential, estimated to be 10 mV, was subtracted from the measurements. Chloride equilibrium potential was calculated to be −60.3 mV.

Sniffer-patch recordings

Transverse hypothalamic slices from *Vgat*^{Cre}::*Npy*^{hrGFP} mice bilaterally injected with AAV2-EF1a-DIO-ChR2(E123T/T159C)-mCherry into the DVC were cut at 200-250 using a Leica VT1200S vibratome. Slices were incubated for one hour in a solution containing (in mM): 124 NaCl, 3 KCl, 1 $CaCl_2$, 3 $MgCl_2$, 26 $NaHCO_3$, 1.25 NaH_2PO_4, 10 D-glucose, and bubbled with 95/5% O_2/CO_2, pH 7.4. After incubation, slices were transferred to a recording chamber continuously superfused with an external solution. The external solution composition differed from incubation solution in containing 2 mM $CaCl_2$ and 2 mM $MgCl_2$.

In all experiments the intracellular pipette solution for voltage-clamp recordings contained (mM): 117.5 Cs-gluconate, 17.5 CsCl, 10 KOH-HEPES, 10 BAPTA, 8 NaCl, 5 QX-314, 2 Mg-ATP, 0.3 GTP; for current-clamp recordings: 126 K-gluconate, 4 NaCl, 5 HEPES, 15 glucose, 1 $MgSO_4 \cdot 7H_2O$, 2 BAPTA, 3 Mg-ATP (pH 7.2, 295-310 mOsm in both cases); pipette resistance was 7-9 MOhm; recordings were performed at 33-35°C using Multiclamp-700B amplifier with -60 or -70 mV holding current (for voltage-clamp recordings); signals were pre-filtered and digitized at 10 kHz. In experiments where transmembrane currents were recorded in outside-out patches only (sniffer-patch recordings), the $GABA_A$ receptors response was isolated with a ligands cocktail containing 50 μM APV, 20 μM NBQX, 50 nM CGP-55845, 200 μM S-MCPG, 10 μM MDL-72222, and 1 μM strychnine.

QUANTIFICATION AND STATISTICAL ANALYSIS

Statistical analyses were performed using GraphPad Prism 9 software. Statistical tests and values are provided in the figure legends. Two-tailed paired or unpaired Student t-test were used when comparing 2 groups and repeated measures (RM) and/or two-way ANOVA with Bonferroni post-hoc correction was used when comparing 4 groups. Nuc-Seq data were analyzed as described above. Randomization and blinding was performed for histological quantifications and where possible for *in vivo* studies. Statistical significance was defined as $p \leq 0.05$. Raw data are stored in Excel spreadsheets and figures have been assembled with CorelVector and Affinity Designer software.

Current Biology, Volume *34*

Supplemental Information

A brainstem to hypothalamic arcuate nucleus GABAergic circuit drives feeding

Pablo B. Martinez de Morentin, J. Antonio Gonzalez, Georgina K.C. Dowsett, Yuliia Martynova, Giles S.H. Yeo, Sergiy Sylantyev, and Lora K. Heisler

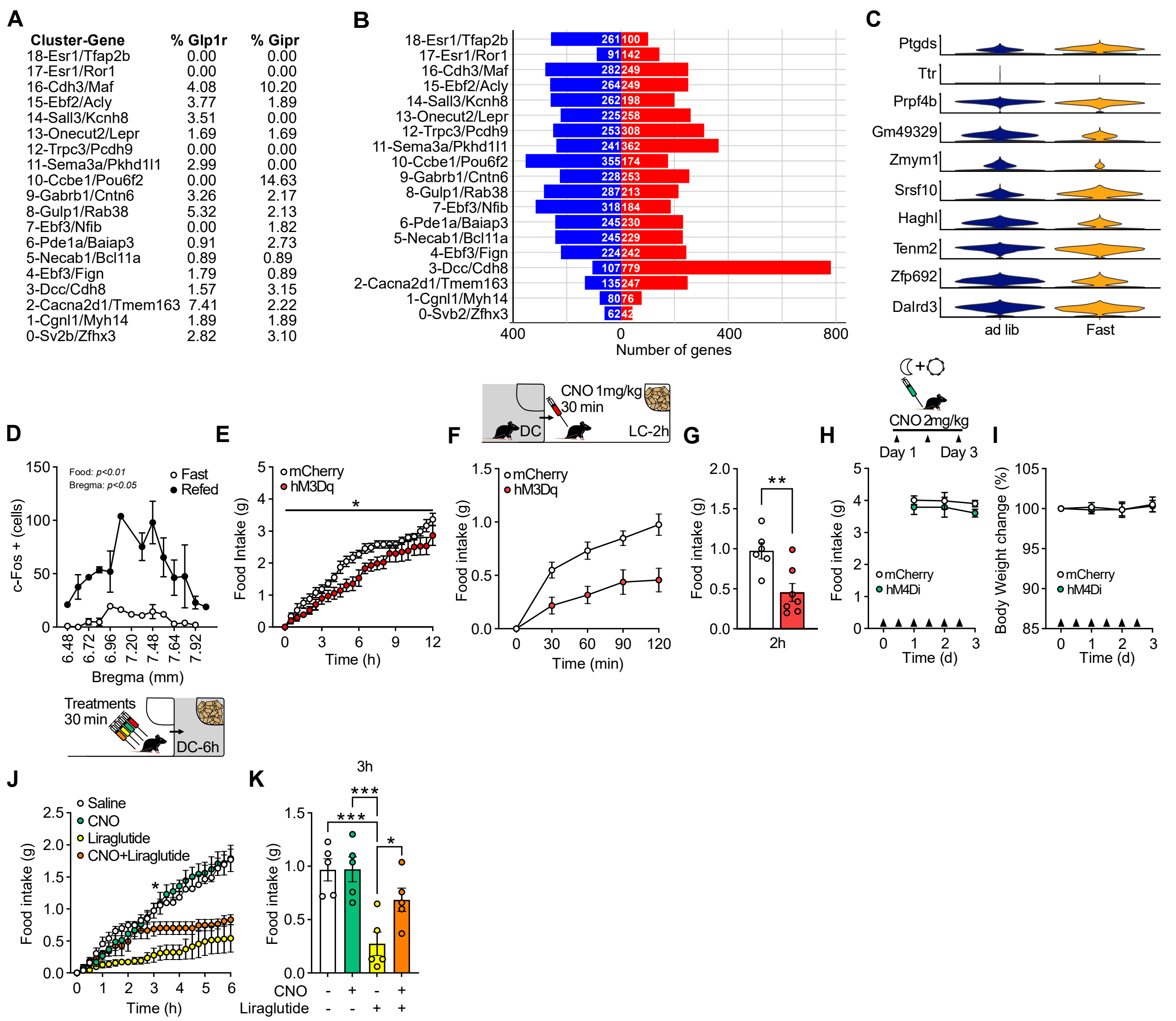

Figure S1. Characterization and function of GABA^{DVC} cells. Related to Figure 1.
(A) Proportion of GLP1-R and GIPR expression in each Slc32a1+ (GABA^{DVC}) cluster. (B) Number of genes upregulated (red) and downregulated (blue) in response to an overnight fast in each cluster. Genes included were significantly differentially regulated (p<0.05). (C) Top 10 genes differentially expressed in *ad libitum* fed vs fasted in Slc32a1+ neurons. (D) Quantification of total NTS c-Fos-expressing cells following 16h fasting and fasting+2h refeeding (n=3, two-way ANOVA Bregma level: $F_{(13,34)}$=3.16, p=0.004; Nutritional state: $F_{(1,4)}$=18.61, p=0.012). (E) 12h dark cycle food intake in GABA^{DVC}:hM3Dq vs control GABA^{DVC}:mCherry mice injected with clozapine-n-oxide (CNO). (F) Food intake following an overnight fast in GABA^{DVC}:hM3Dq mice compared to control GABA^{DVC}:mCherry (n=6, RM two-way ANOVA $F_{(1,11)}$=10.97, p=0.0069) treated with CNO (1 mg/kg, i.p.) and represented as (G) 2h total food intake (n=6/7, Unpaired t test, t(11)=3.392, p=0.006). (H) Food intake and (I) body weight change in GABA^{DVC}:hM4Di vs control GABA^{DVC}:mCherry mice injected twice daily with CNO (2 mg/kg, i.p.). (J-K) Food intake in GABA^{DVC}:hM4Di mice co-administered with CNO and Liraglutide (RM: ANOVA $F_{(3,12)}$=15.64, p=0.002, Bonferroni adjusted p=0.0004 Saline vs Liraglutide, P=0.0004 CNO vs Liraglutide and P=0.0263 Liraglutide vs Liraglutide+CNO). *: p<0.05, **: p<0.01, ***: p<0.001.

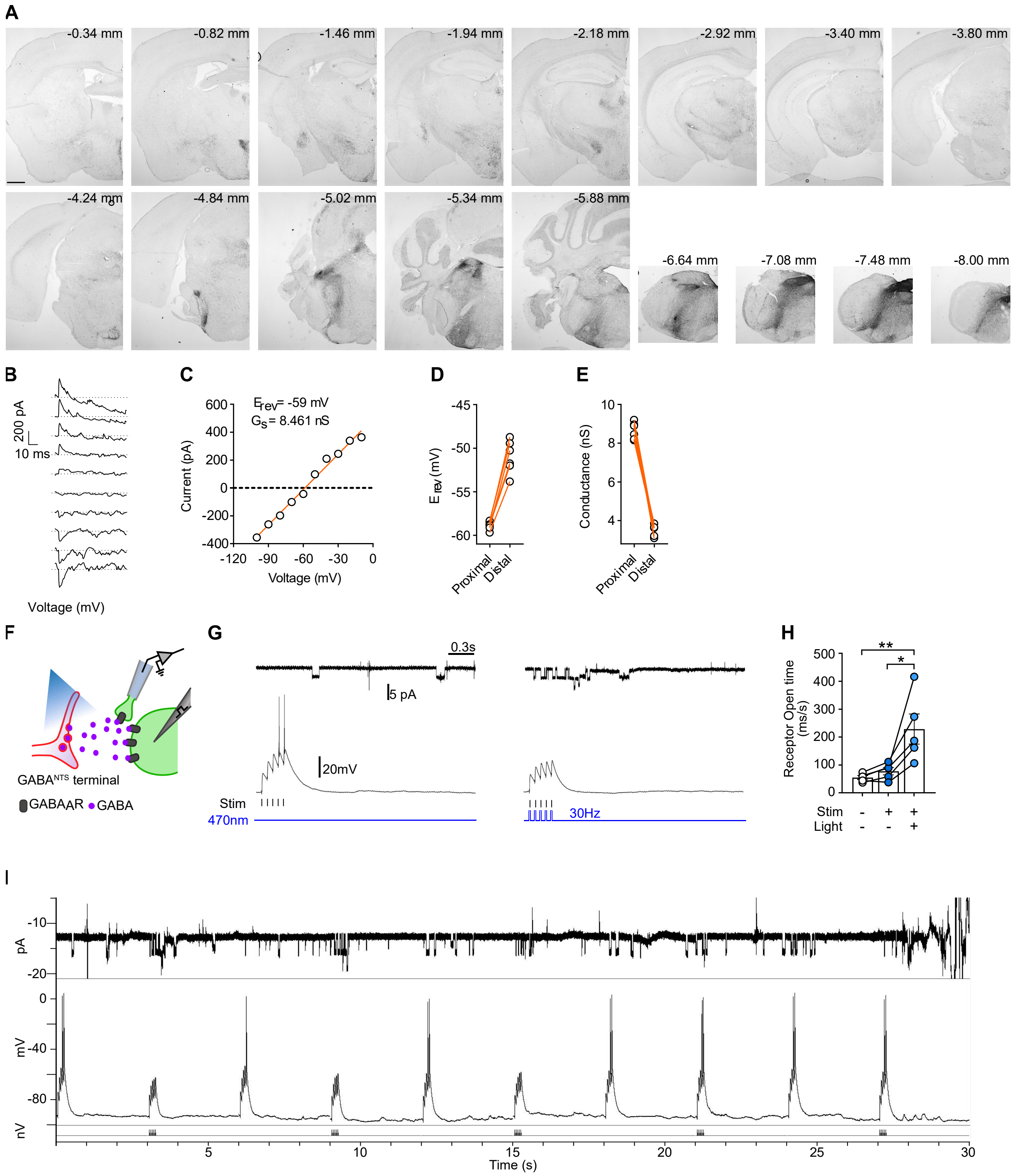

Figure S2. GABA^{DVC} projection pattern and CRACM additional analysis. Related to Figure 2.

(A) Representative photomicrographs (scalebar 100 μm) of ChR2-mCherry fibers in a serial rostro-caudal distribution of a *Vgat^{Cre}* mouse brain injected with AAV-DIO-ChR2-mCherry into the DVC. (B-E) A simulated voltage-clamp experiment in an anatomically realistic model of a medium spiny neuron, using the same voltage steps as those described for electrophysiology experiments (Figure 2E-G). The effect of optogenetic stimulation was modelled as a single GABAergic event taking place at each step. (C) Current amplitude was plotted against

voltage (as in Figure 2E-G), and a linear fit was used to estimate peak conductance G_s and reversal potential E_{rev}. (D-E) Simulated optogenetic activation of GABA inputs making synaptic contact only on dendrites close to the soma ("prox.", proximal dendrites) was compared to the same type of activation of GABA synapses connected only to dendrites far from the soma ("distal" dendrites) in six different medium spiny neuron models. The reversal potential for GABA in the model was set to -60 mV, and this is close to what was measured at the soma when GABA synaptic inputs were located on dendrites close to the soma. However, when GABA synapses were activated only on dendrites distant from the soma E_{rev}, was more positive than expected and conductance G_s was attenuated. (F) Diagram of outside-out patch technique coupled to a postsynaptic electrical stimulator. (G, left) Representative response of NPY^{hrGFP} cell from $Vgat^{Cre}$::Npy^{hrGFP} mouse injected with AAV-DIO-ChR2-mCherry into the NTS subjected to series of five electrical stimuli, each evoking excitatory post-synaptic potential (EPSP) of sub-threshold amplitude. (G, right) The same cell with electrical stimuli coupled with 470 nm light burst. (H) Quantification of receptor opening time (n=5, two-way RM ANOVA $F_{(2,8)}=11.28$; p=0.0047, Bonferroni adjusted P=0.0072 NS vs SL and P=0.0157 S vs SL). (I) Representative response of a single NPY^{hrGFP} cell subjected to 10 series of five electrical stimuli, each evoking excitatory post-synaptic potential (EPSP) of sub-threshold amplitude coupled with 470nm light burst in $Vgat^{Cre}$::Npy^{hrGFP} mouse injected with AAV-DIO-ChR2-mCherry into the NTS. NS: No stimulation; S: Stimulation; SL: Stimulation + Light. *: p<0.05, **: p<0.01.

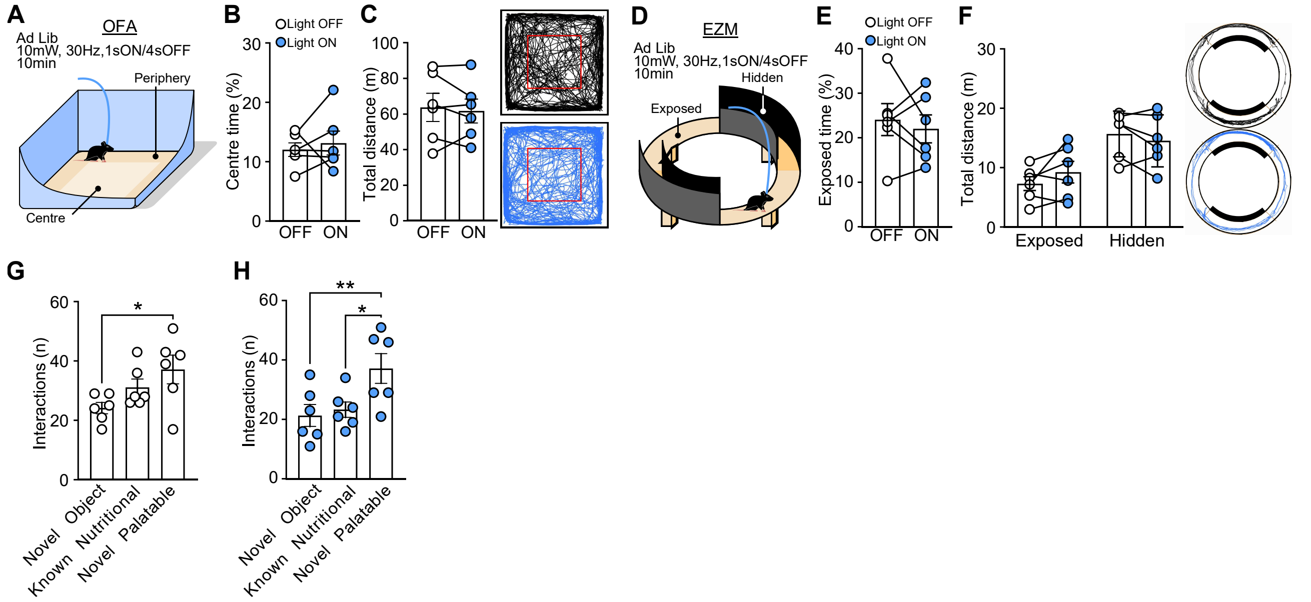

Figure S3. Optogenetic activation of GABA^{DVC}→ARC in VgatCre mice does not induce anxiety-like behavior. Related to Figure 3.

(A) Diagram illustrating open field arena (OFA) task. GABA^{DVC}→ARC optogenetic activation does not alter (B) time spent in the center or (C) distance travelled (representative trace of the movement) during the test. (D) Diagram illustrating elevated zero maze (EZM) task. GABA^{DVC}→ARC optogenetic activation does not alter (E) time spent in the exposed zone or (F) distance travelled in each zone (representative trace of the movement) during the test. (G-H) Quantification of number of interactions with a novel object, a known nutritional food item or a novel palatable food item in mice (G) without GABA^{DVC}→ARC stimulation (RM ANOVA ($F_{(2,10)}$=4.993; p=0.0314, Bonferroni adjusted p=0.0306 novel object vs novel palatable object) and (H) with GABA^{DVC}→ARC stimulation (RM ANOVA $F_{(2,10)}$=10.71; p=0.0033, Bonferroni adjusted p=0.0051 novel object vs novel palatable object; p=0.0121 known nutritional object vs novel palatable object). n=6 mice. *:p<0.05; **:p<0.01.
